# Comprehensive investigation of the prognostic values and molecular mechanisms of syntaxin binding protein 5 antisense RNA 1 in patients with colon adenocarcinoma based on RNA sequencing dataset

**DOI:** 10.7150/jca.83423

**Published:** 2023-06-04

**Authors:** Haotang Wei, Li Tang, Jialei Wang, Min Ni, Xiwen Liao, Erna Guo

**Affiliations:** 1 Department of Gastrointestinal Surgery, The Third Affiliated Hospital of Guangxi Medical University, 530031, Guangxi Zhuang Autonomous Region, People's Republic of China.; 2 Department of Hepatobiliary Surgery, The First Affiliated Hospital of Guangxi Medical University, Nanning, 530021, Guangxi Zhuang Autonomous Region, People's Republic of China.; 3 School of Public Health, Guangxi Medical University, Nanning, 530021, Guangxi Zhuang Autonomous Region, People's Republic of China.; 4 Institute of International Education, Guangxi Medical University, Nanning, 530021, Guangxi Zhuang Autonomous Region, People's Republic of China.

**Keywords:** syntaxin binding protein 5 antisense RNA 1, colon adenocarcinoma, molecular mechanism, prognostic value, The Cancer Genome Atlas

## Abstract

**Objective:** The main purpose of this study is to perform a comprehensive investigation of the prognostic value and molecular mechanism of syntaxin binding protein 5 antisense RNA 1 (STXBP5-AS1) through the whole genome RNA sequencing data of the The Cancer Genome Atlas (TCGA) colon adenocarcinoma (COAD) cohort.

**Methods:** There were 438 COAD patients were fit into current study for survival analysis. Gene expression profiling interactive analysis 2.0, Database for Annotation, Visualization and Integrated Discovery v6.8, gene set enrichment analysis (GSEA) and connectivity map (CMap) are used to investigate the molecular mechanisms and targeted drugs of STXBP5-AS1 in COAD.

**Results:** By comparing the expression level of tumor and non-tumor tissues, we found that STXBP5-AS1 was notablely down-regulated in COAD tumor tissues. Survival analysis suggested that low STXBP5-AS1 expression was significantly related to poor overall survival (OS) of COAD (log-rank P=0.035, adjusted P=0.005, HR=0.545, 95%CI=0.356-0.836). The enrichment analysis of STXBP5-AS1 co-expressed genes, GSEA and differentially expressed genes suggests that STXBP5-AS1 may play a part in COAD by regulating the following biological processes or pathways: cell junction, DNA replication, apoptosis, cell cycle, metastasis, tumor protein 53, Wnt, mTORC1, MCM, notch receptor 4, transforming growth factor beta receptor, and cGMP-PKG signaling pathway. CMap analysis was screened out four small molecule drugs (anisomycin, cephaeline, NU-1025 and quipazine) that may be used as STXBP5-AS1 targeted therapy drugs in COAD. The co-expression analysis of STXBP5-AS1 and immune cell gene signature indicated that STXBP5-AS1 was significantly related to immune cell gene set in normal intestinal tissues, but not in COAD tumor tissues.

**Conclusion:** Our results revealed that STXBP5-AS1 is notablely down-regulated in COAD tumor tissues, and may act as a novel prognostic biomarker for COAD.

## Introduction

Colon adenocarcinoma (COAD) is the most common pathological type of colon cancer, and its main treatment strategy is surgery. The etiology of COAD remains to be explored, but the occurrence of this disease is closely related to a diet with more fat and less fiber, and is similar to other solid tumors, driven by environmental and genetic factors. The Cancer Genome Atlas (TCGA) is an open access database that provides whole-genome and multi-omics data sets of a variety of cancers for researchers to download and use 1. The COAD whole-genome RNA sequencing (RNA-seq) data set include into current study for survival analysis and mechanism investigation was download from TCGA Data Portal. Long non-coding RNA (lncRNA) has been widely published to be closely related to cancer occurrence, development and prognosis, and can be used as a biomarker for cancer diagnosis, prognosis monitoring, recurrence and metastasis, including colon cancer 2, 3. Previous studies have suggested that STXBP5-AS1 is closely related to a variety of cancers and acts as tumor suppressor gene in cancers 4-6. However, by reviewing the literature, we have not found any reports on the clinical application value and molecular mechanism of STXBP5-AS1 in colorectal cancer. In order to compensate for these research gaps, the main purpose of this study is to conduct a comprehensive investigation of the prognostic value and molecular mechanism of STXBP5-AS1 through the whole genome RNA-seq data of the TCGA COAD cohort.

## Materials and methods

### Data downloading and preprocessing

The level 3 RNA sequencing dataset and corresponding clinical dataset were downloaded from the TCGA Data Portal (https://portal.gdc.cancer.gov/) 7. The RNA sequencing dataset was normalized using the *edgeR* package 8. We merged RNA sequencing dataset and clinical dataset, and finally included 438 COAD patients with both RNA sequencing dataset and clinical prognostic parameters. The clinical parameters included in this study are age, gender and tumor stage. Since the authors of this study did not perform any experiments involving animal or human tissues, this study does not require additional ethics committee approval. At the same time, the acquisition, use and release of the COAD dataset of this study are in compliance with the TCGA guidelines. The present study was approved by the Ethics Committee of The Third Affiliated Hospital of Guangxi Medical University (also known as The Second Nanning People's Hospital)**,** and the approval number is Y2021099.

### Clinical significance investigation of STXBP5-AS1 in THCA

The expression distribution of STXBP5-AS1 in the TCGA pan-cancer cohort comes from the online analysis tool, which called gene expression profiling interactive analysis 2.0 (GEPIA 2.0, http://gepia2.cancer-pku.cn/#index) 9. The expression distribution of STXBP5-AS1 in the TCGA COAD and GTEx normal intestinal tissue cohorts were also derived from the GEPIA 2.0 online analysis tool. The relationship between STXBP5-AS1 and tumor immune cell gene signatures also can be implemented on GEPIA 2.0. Using receiver operating characteristic (ROC) curve method to evaluate the ability of STXBP5-AS1 mRNA expression to identify COAD tumor and adjacent normal tissues. Cox proportional hazards regression model and nomogram model were used to evaluate the prognostic value of STXBP5-AS1 for COAD overall survival (OS). The nomogram model is drawn in the R platform by the *rms* package. Joint effect survival analysis was used to evaluate the ability of STXBP5-AS1 combined with clinical parameters to predict the OS of COAD.

### Functional enrichment of STXBP5-AS1 in COAD

We screened STXBP5-AS1 co-expressed protein coding genes (PCGs) by using the whole genome RNA sequencing dataset through the *Cor* function in R platform. Co-expression correlation relationship is evaluated using Pearson's correlation coefficient, and *P* value <0.05 is considered as statistically significant. Functional enrichment analysis of STXBP5-AS1 co-expressed genes was carried out using the Database for Annotation, Visualization and Integrated Discovery (DAVID) v6.8 (https://david.ncifcrf.gov/home.jsp) 10, 11, which included Gene Ontology (GO) term and Kyoto Encyclopedia of Genes and Genomes (KEGG) analysis. Subsequently, we also used the gene set enrichment analysis (GSEA) method to perform differential functional enrichment analysis between patients with high and low STXBP5-AS1 phenotypes 12, 13. To achieve statistical significance, the GSEA analysis result must meet the following conditions: |normalized enrichment score (NES)|> 1, nominal *P* < 0.05 and false discovery rate (FDR) < 0.25. Subsequently, we also used *edge*R to screen the differentially expressed genes (DEGs) between patients with high and low STXBP5-AS1 phenotypes for functional enrichment analysis. Genes that meet the following conditions are considered DEGs: |log2 fold change (FC)| > 1, *P* value < 0.05 and FDR < 0.05. The prognostic analysis of STXBP5-AS1 co-expressed genes and DEGs are calculated in the R platform using the* survival* package. STXBP5-AS1 targeted therapy drugs were screened through connectivity map (CMap, https://portals.broadinstitute.org/cmap/) 14, 15, and the drugs' chemical structure and drug-gene interaction networks were derived from PubChem (https://pubchem.ncbi.nlm.nih.gov/) and STITCH (http://stitch.embl.de/cgi/), respectively 16-19.

### Statistical analysis

Kaplan-Meier survival analysis uses log-rank test, hazard ratio (HR) and 95% confidence interval (CI) are used to evaluate the prognostic difference in the Cox proportional hazards regression model. *P* value < 0.05 is considered as statistically significant. All statistical analyses were performed by SPSS version 26.0 (IBM Corporation, Armonk, NY, USA) and R version 3.6.2.

## Results

### Clinical significance investigation of STXBP5-AS1

**Figure 1:**
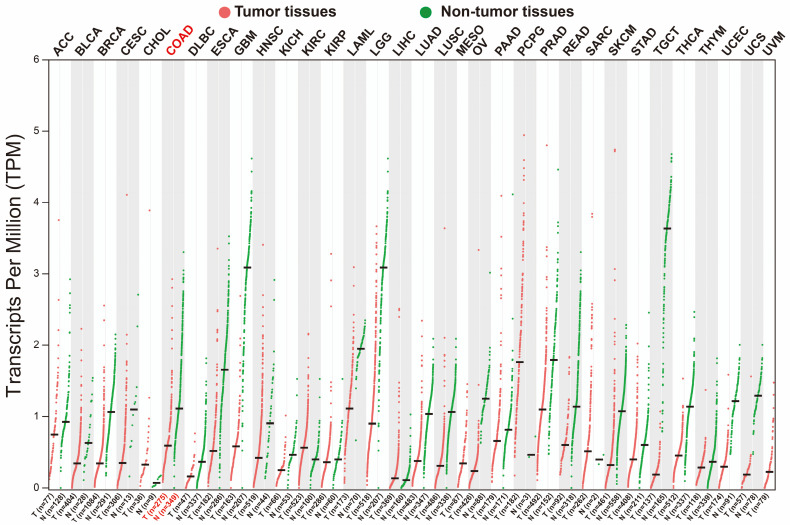
Scatter plot of the expression distribution of STXBP5-AS1 in TCGA pan-cancer cohort.

**Figure 2:**
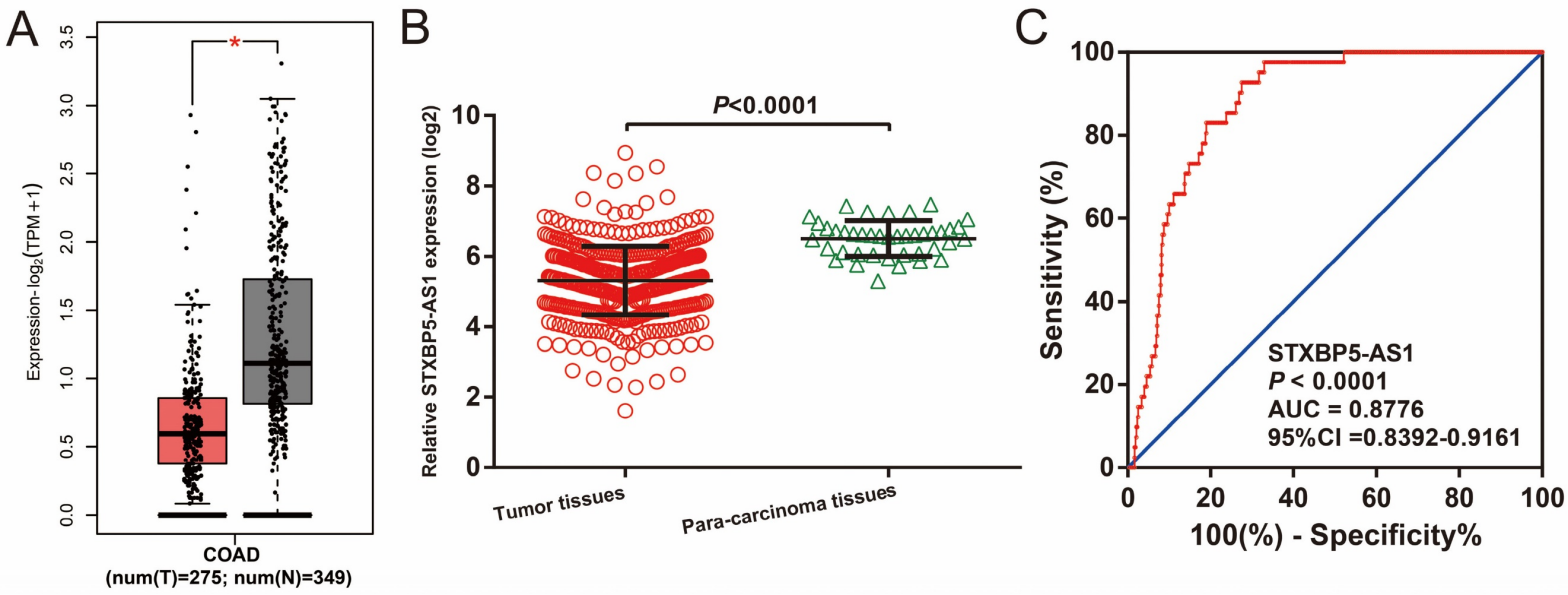
The expression distribution of STXBP5-AS1 in COAD tumor and non-tumor tissues. (A) Box plot of STXBP5-AS1 in COAD tumor and non-tumor tissues; (B) Scatter plot of STXBP5-AS1 in COAD tumor and para-carcinoma tissues; (C) The ROC curve of STXBP5-AS1 in differentiating COAD tumor and para-carcinoma tissues.

**Figure 3:**
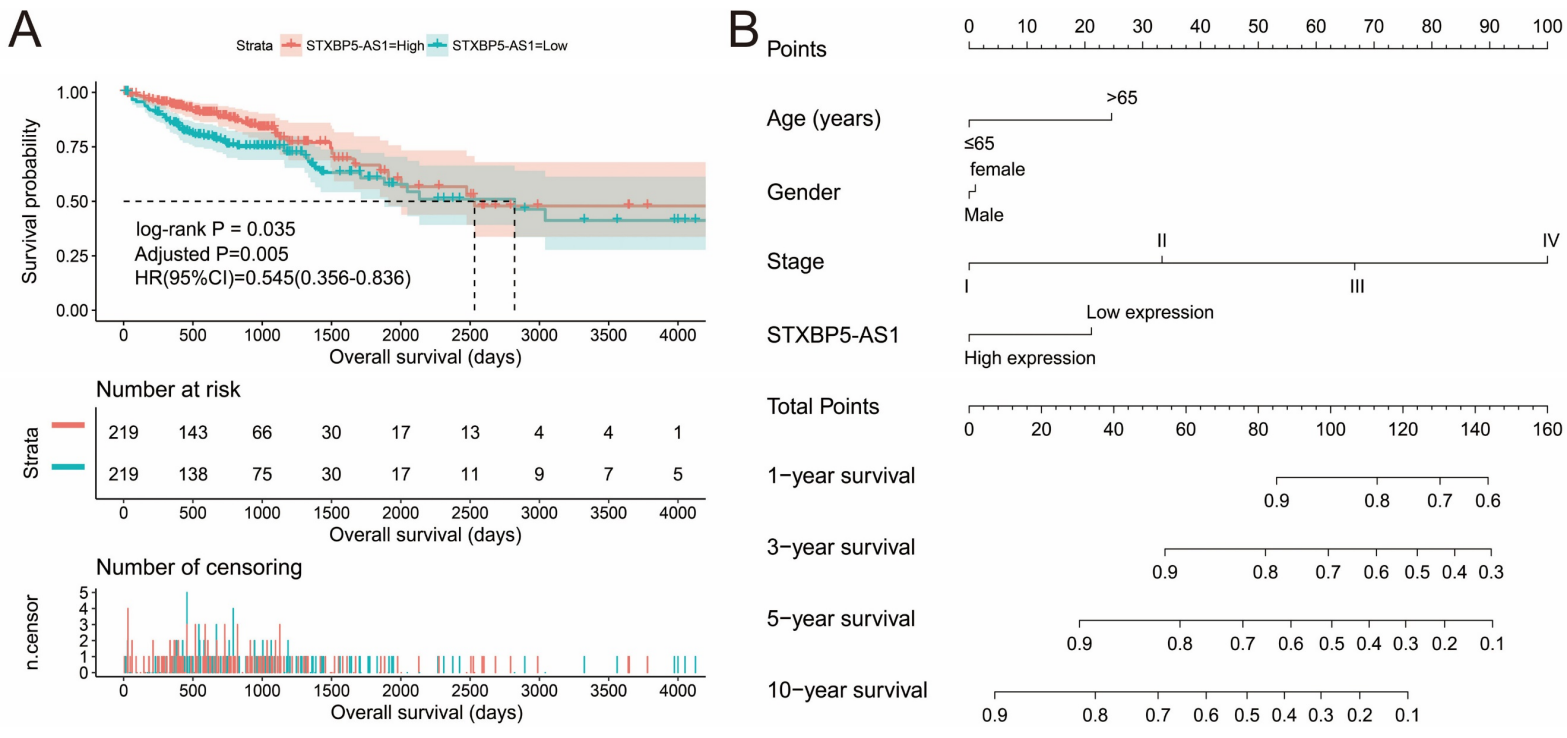
Prognostic value of STXBP5-AS1 in TCGA COAD cohort. (A) Kaplan-Meier survival curve of STXBP5-AS1 in TCGA COAD cohort; (B) Nomogram of STXBP5-AS1 in TCGA COAD cohort.

**Figure 4:**
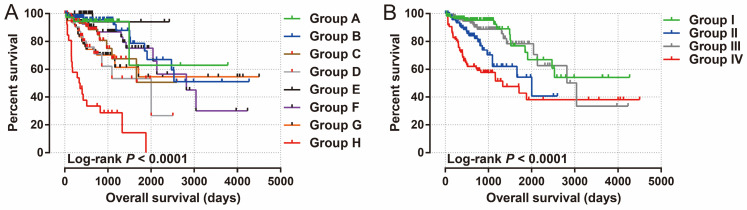
Joint effects survival analysis of tumor stage and the STXBP5-AS1 expression with OS in COAD patients. Stratified by STXBP5-AS1 expression and tumor stage (A-B).

We used GEPIA2.0 online tool to generate the expression distribution of STXBP5-AS1 in TCGA pan-cancer cohort and found that STXBP5-AS1 was notablely up-regulated in most cancers' tumor tissues (**Figure 1**). By comparing the expression level of STXBP5-AS1 between COAD tumor and non-tumor tissues, we found that STXBP5-AS1 was notablely down-regulated in COAD tumor tissues (**Figure 2A-B**). ROC analysis suggests that the mRNA expression of STXBP5-AS1 can notablely differentiate COAD tumor tissues from adjacent non-tumor tissues (**Figure 2C**, AUC = 0.8776, 95%CI = 0.8392-0.9161). The clinical parameters of COAD are summarized in **Table S1**. Survival analysis suggested that low STXBP5-AS1 expression was significantly associated with poor overall survival (OS) of COAD (log-rank P = 0.035, adjusted P = 0.005, HR = 0.545, 95%CI = 0.356-0.836,** Figure 3A**). The nomogram suggests that STXBP5-AS1 has a certain contribution to the prognosis of COAD. Among the clinical parameters, tumor stage contributes the most to the prognosis of COAD in this cohort (**Figure 3B**). Joint effects survival analysis of tumor stage and the STXBP5-AS1 expression in COAD OS were summarized in** Table 1** and **Figure 4A-B**. The joint effects survival analysis suggests that STXBP5-AS1 can classify the COAD subgroup patients with significant differences in prognosis in a more detailed manner.

### Functional enrichment of STXBP5-AS1 in COAD

**Figure 5:**
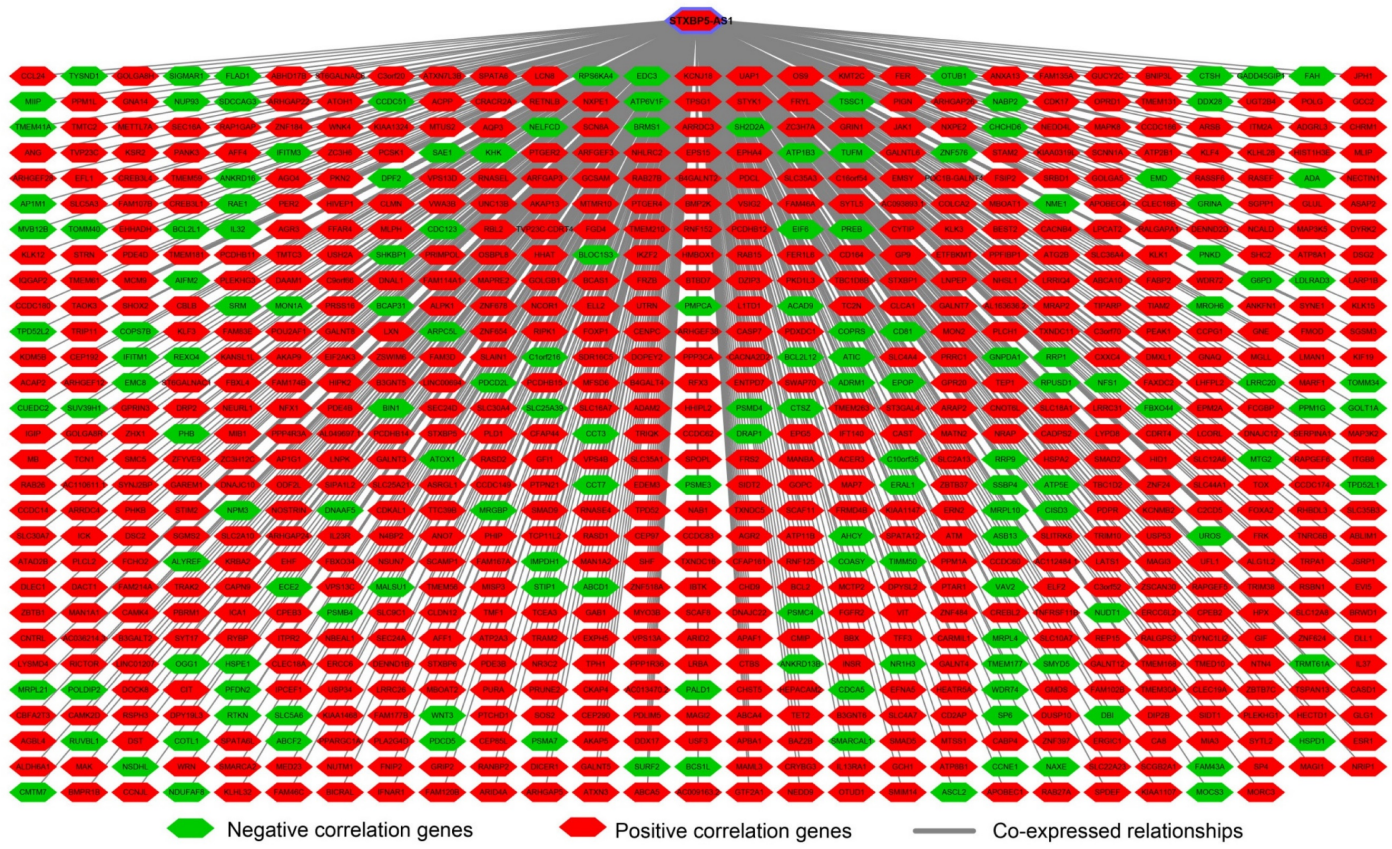
Interaction network plot of STXBP5-AS1 and its co-expressed PCGs.

**Figure 6:**
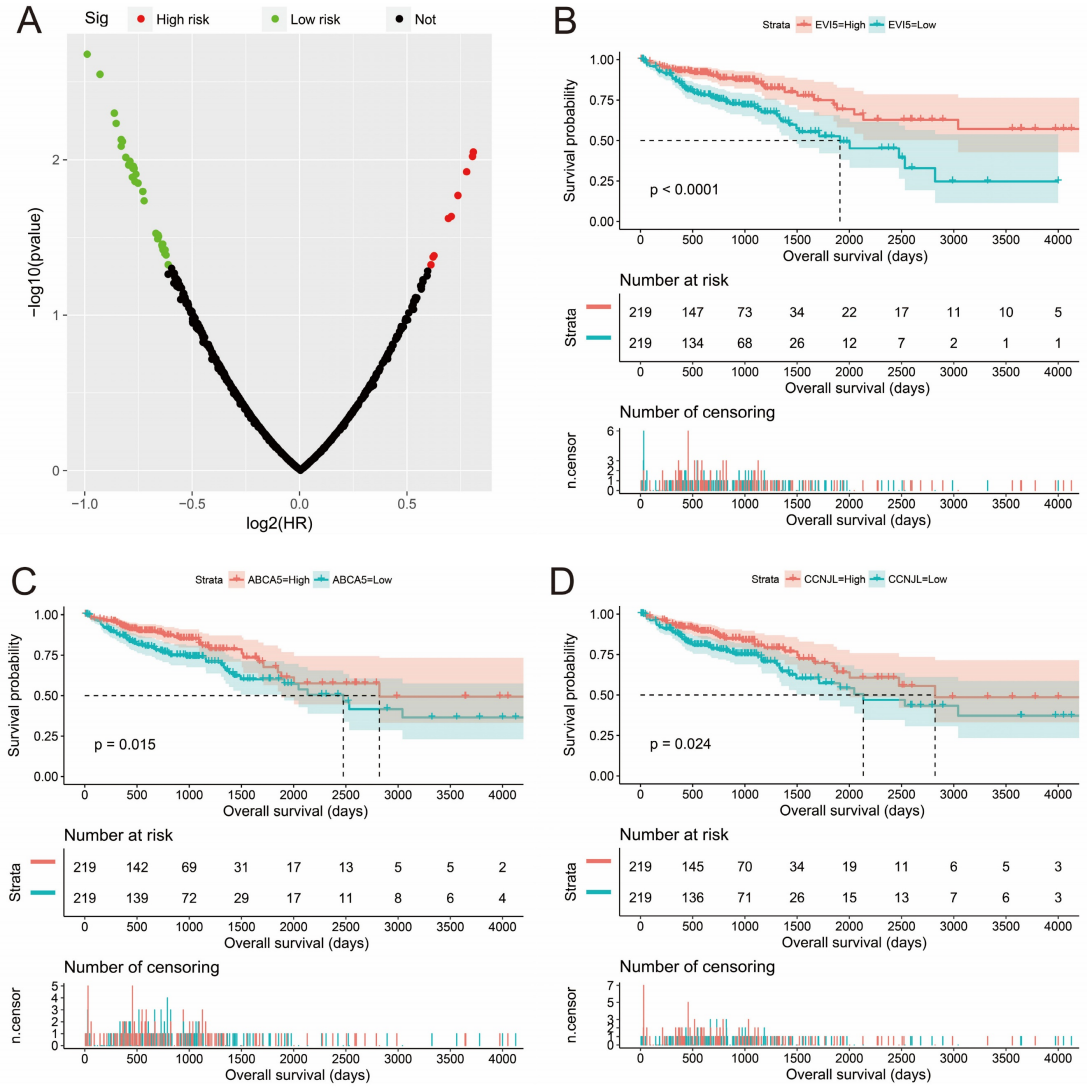
Survival analysis results of STXBP5-AS1 co-expressed PCGs. (A) Volcano plot of STXBP5-AS1 co-expressed PCGs survival analysis results; (B) Kaplan-Meier curve of EVI5; (C) Kaplan-Meier curve of ABCA5; (D) Kaplan-Meier curve of CCNJL.

To understand the biological function of STXBP5-AS1 in COAD, we used whole-genome RNA sequencing dataset to screen co-expressed PCGs of STXBP5-AS1. A total of 781 STXBP5-AS1 co-expressed PCGs are obtained, of which 153 are negatively related PCGs and 628 are positively related PCGs (**Figure 5, Table S2**). Function enrichment of STXBP5-AS1 co-expressed PCGs suggest that STXBP5-AS1 are significantly involved in biological processes and pathways such as mucin type O-Glycan biosynthesis, cAMP signaling pathway, cGMP-PKG signaling pathway, O-glycan processing, Rho guanyl-nucleotide exchange factor activity, positive regulation of apoptotic process, transforming growth factor beta receptor, pathway-specific cytoplasmic mediator activity, activation of Jun kinase activity, SMAD binding, SMAD protein signal transduction, cell junction, SMAD protein complex in COAD (**Table S3**). Subsequently, we performed multivariate survival analysis on these STXBP5-AS1 co-expressed PCGs in the R platform, and we identified 42 PCGs that are significantly related to the OS of COAD (**Table S4, Figure 6A**). The top three significance PCGs were ecotropic viral integration site 5 (EVI5, log-rank P < 0.0001, adjusted P = 0.002, HR = 0.503, 95%CI = 0.324-0.779, **Figure 6B**), ATP binding cassette subfamily A member 5 (ABCA5, log-rank P = 0.015, adjusted P = 0.003, HR = 0.524, 95%CI = 0.343-0.801, **Figure 6C**) and cyclin J like (CCNJL, log-rank P = 0.024, adjusted P = 0.005, HR = 0.549, 95%CI = 0.361-0.835, **Figure 6D**).

**Figure 7:**
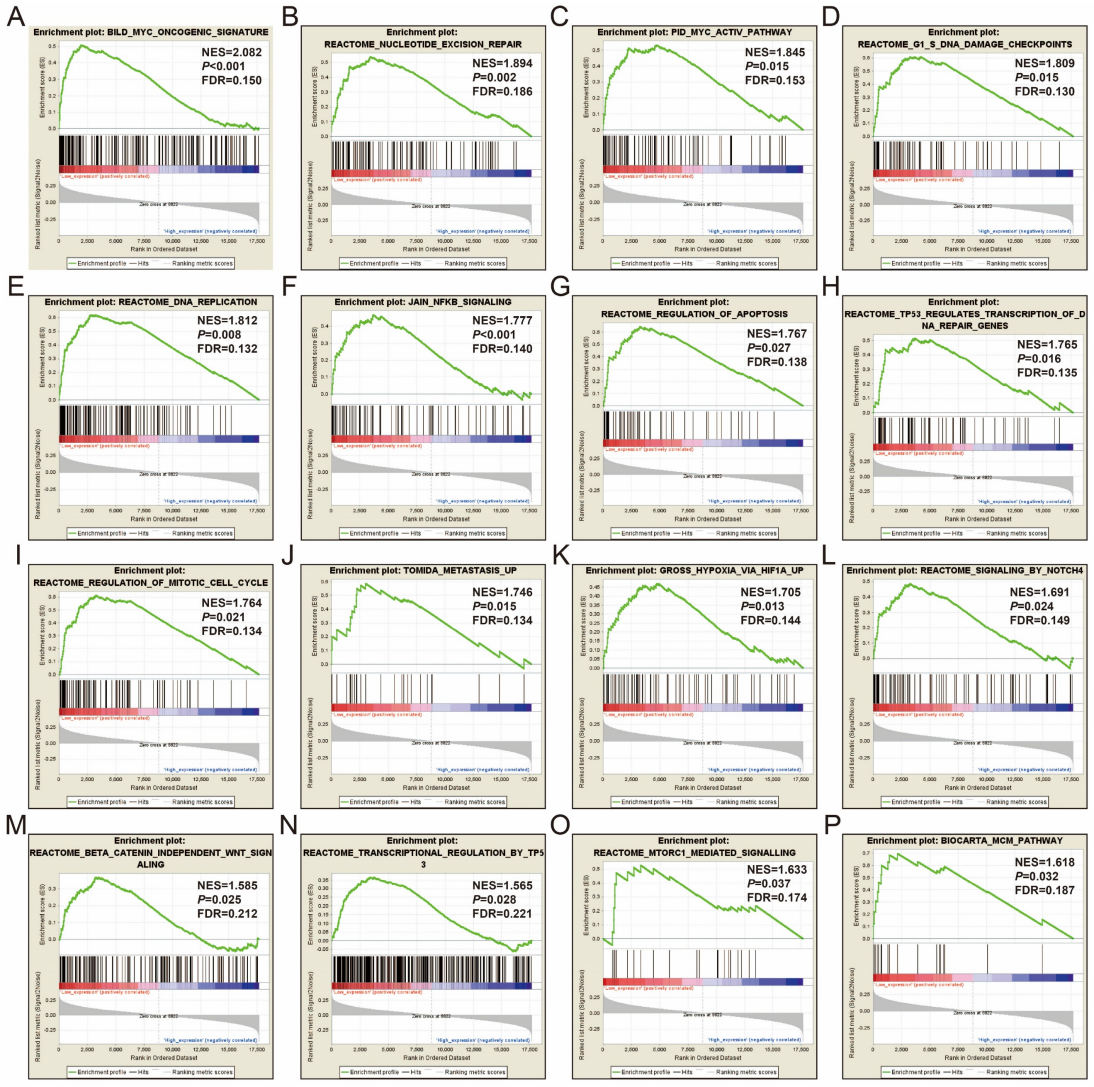
GSEA analysis between low- and high-STXBP5-AS1 phenotypes using the c2 reference gene set. (A) Myc oncogenic signature, (B)nucleotide excision repair, (C)Myc active pathway, (D) G1/S DNA damage checkpoints, (E) DNA replication, (F) JAIN/NFKB signaling, (G) regulation of apoptosis, (H) TP53 regulates transcription of DNA repair genes, (I) regulation of mitotic cell cycle, (J) metastasis up, (K) hypoxia via HIF1A up, (L) signaling by NOTCH4, (M) beta catenin independent Wnt signaling, (N) transcription regulation by TP53, (O) mTORC1 mediated signaling, (P) MCM pathway.

**Figure 8:**
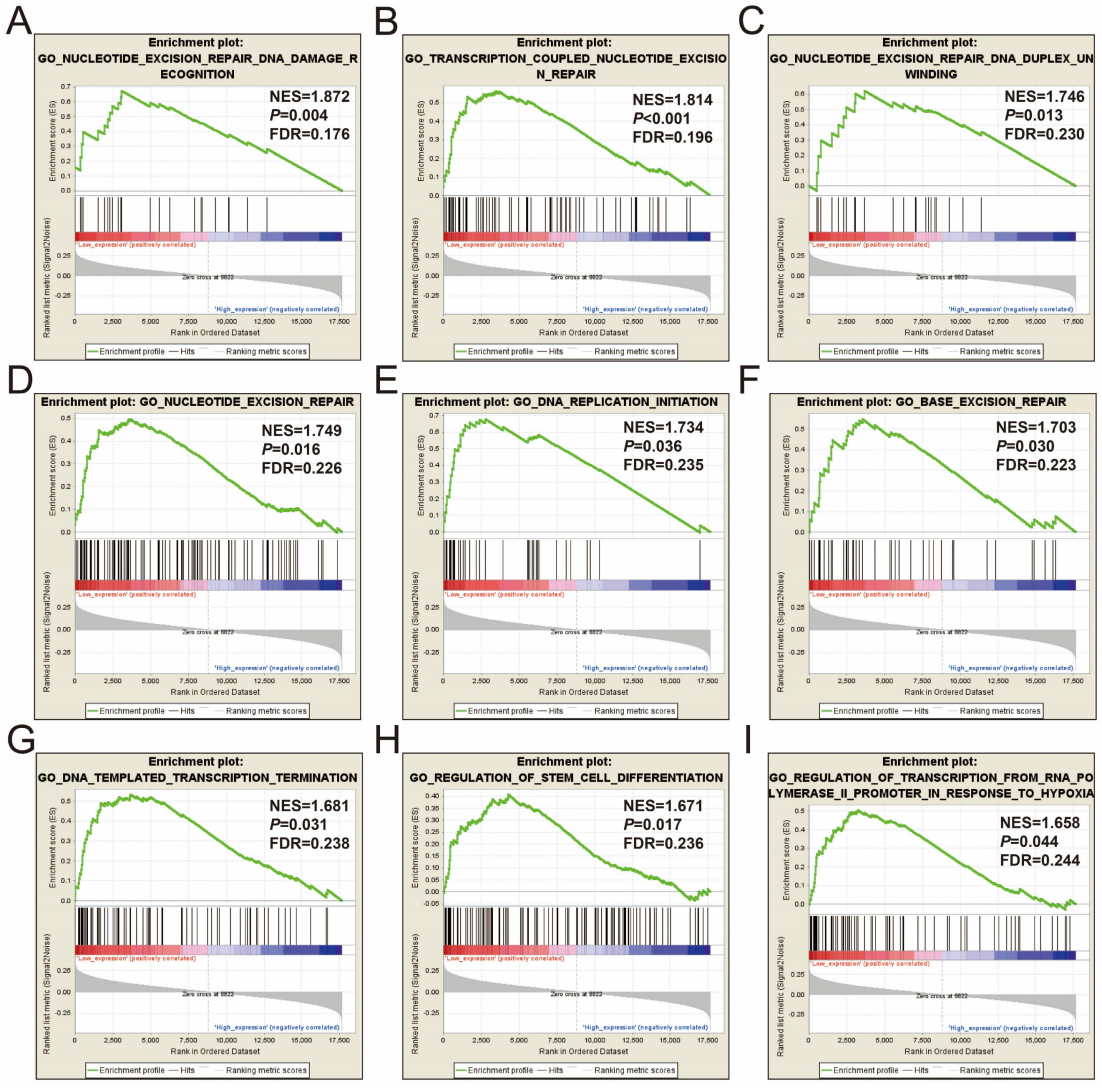
GSEA analysis between low- and high-STXBP5-AS1 phenotypes using the c5 reference gene set. (A) nucleotide excision repair DNA damage recognition, (B) transcription coupled nucleotide excision repair, (C) nucleotide excision repair DNA duplex unwinding, (D) nucleotide excision repair, (E) DNA replication initiation, (F) base excision repair, (G) DNA templated transcription termination, (H) regulation of stem cell differentiation, (I) regulation of transcription from RNA polymerase II promoter in response to hypoxia.

In order to further understand the function of STXBP5-AS1, we used the COAD genome-wide dataset for GSEA analysis, and we found that there are significant differences in the biological function mechanisms between the high-expression- and low-expression-STXBP5-AS1 phenotypes. GSEA analysis using c2 reference geneset suggest that pathways of Myc oncogenic signature, nucleotide excision repair, Myc active pathway, G1/S DNA damage checkpoints, DNA replication, JAIN/NFKB signaling, regulation of apoptosis, TP53 regulates transcription of DNA repair genes, regulation of mitotic cell cycle, metastasis up, hypoxia via HIF1A up, signaling by NOTCH4, beta catenin independent Wnt signaling, transcription regulation by TP53, mTORC1 mediated signaling, and MCM pathway in low-expression-STXBP5-AS1 phenotypes were significant different from high-expression-STXBP5-AS1 phenotypes (**Figure 7A-P, Table S5**). GSEA analysis using c2 reference gene set suggest that biological process of nucleotide excision repair DNA damage recognition, transcription coupled nucleotide excision repair, nucleotide excision repair DNA duplex unwinding, nucleotide excision repair, DNA replication initiation, base excision repair, DNA templated transcription termination, regulation of stem cell differentiation, and regulation of transcription from RNA polymerase II promoter in response to hypoxia in low-expression-STXBP5-AS1 phenotypes were significant different from high-expression-STXBP5-AS1 phenotypes (**Figure 8A-I, Table S6**).

**Figure 9:**
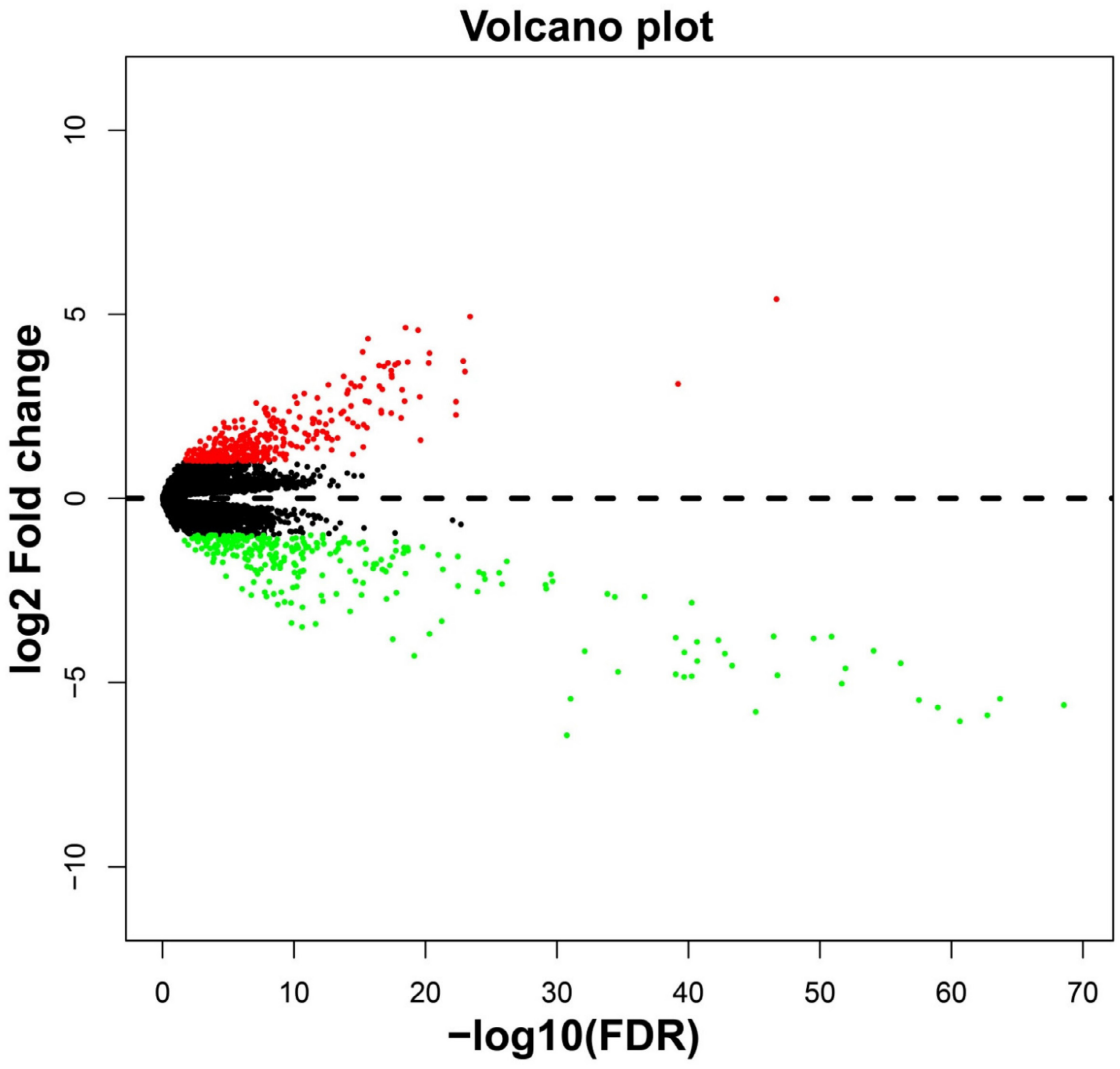
Volcano plot of DEGs high- and low-STXBP5-AS1 phenotypes in COAD.

**Figure 10:**
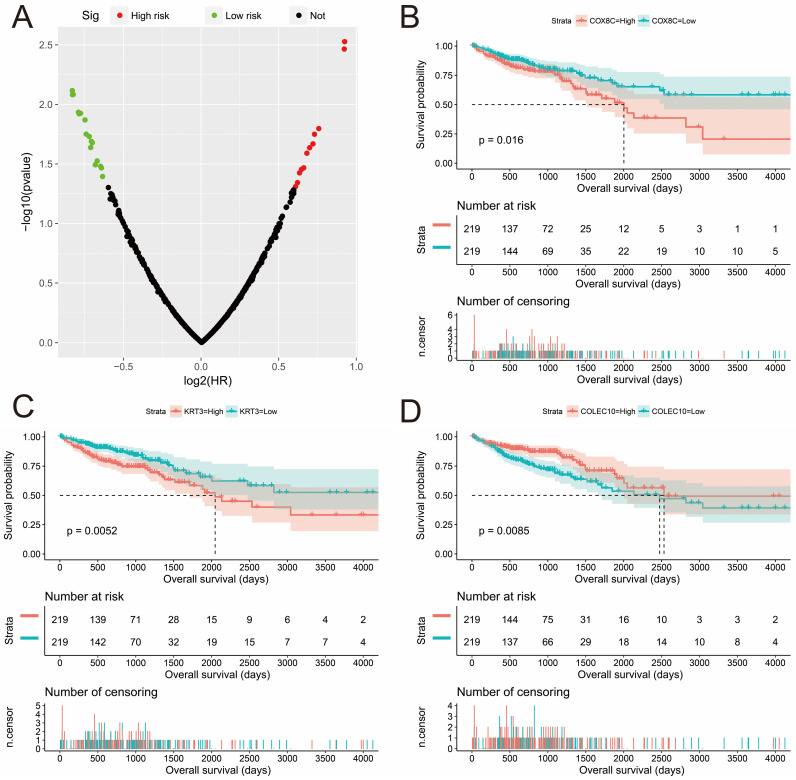
Survival analysis results of DEGs high- and low-STXBP5-AS1 phenotypes in COAD. (A) Volcano plot of survival analysis results of DEGs high- and low-STXBP5-AS1 phenotypes; (B) Kaplan-Meier curve of COX8C; (C) Kaplan-Meier curve of KRT3; (D) Kaplan-Meier curve of COLEC10.

**Figure 11:**
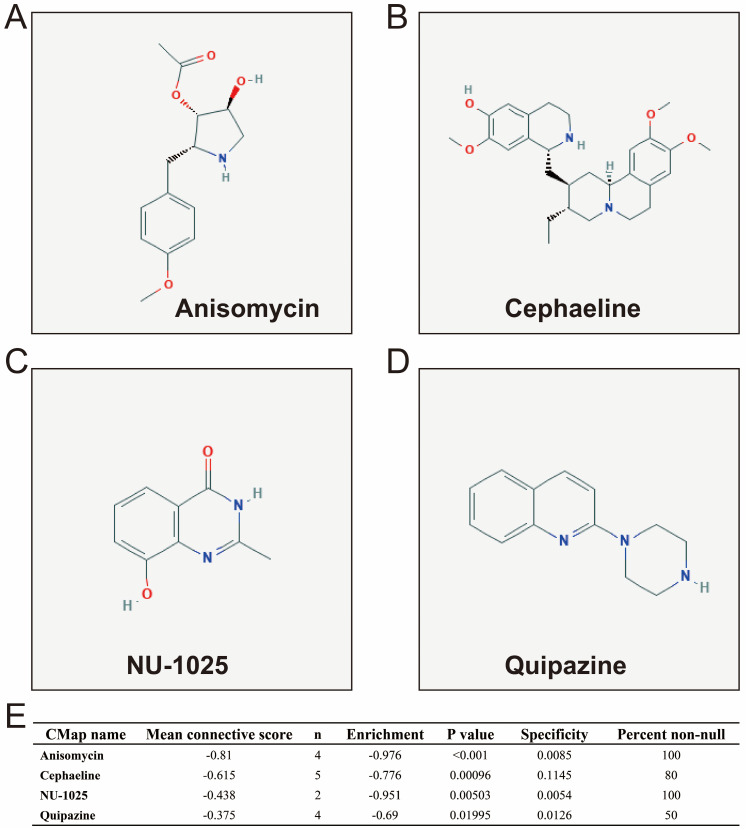
CMap results of STXBP5-AS1 in COAD. (A) Chemical structure of anisomycin; (B) Chemical structure of cephaeline; (C) Chemical structure of NU-1025; (D) Chemical structure of quipazine; (D) CMap results list.

**Figure 12:**
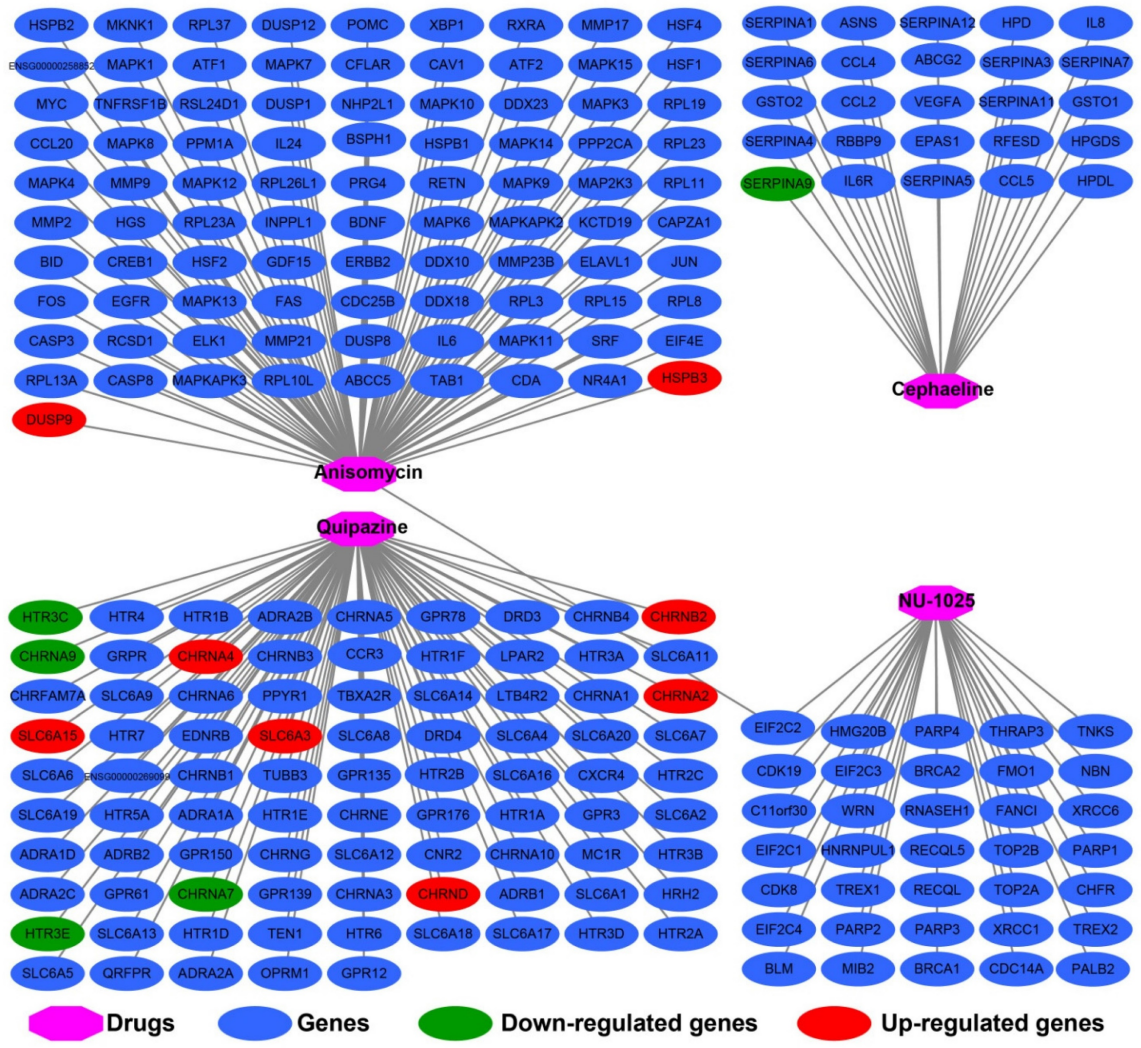
Drug-gene network interaction network plot of the four STXBP5-AS1 targeted small molecule drugs.

In addition to investigating the function of STXBP5-AS1 in COAD through the above two analysis approaches, we also use *edgeR* to screen for DEGs between high- and low-STXBP5-AS1 phenotypes for functional enrichment analysis. We obtained a total of 579 DEGs, of which 317 genes were up-regulated in the low-STXBP5-AS1 phenotype and 262 were down-regulated (**Figure 9, Table S7**). Functional enrichment analysis suggests that these DEGs are significantly involved in the regulation of the following biological functions and pathways: viral carcinogenesis, Transcriptional misregulation in cancer, mucin type O-Glycan biosynthesis, chemical carcinogenesis, DNA replication-dependent nucleosome assembly, negative regulation of megakaryocyte differentiation, DNA replication-independent nucleosome assembly, positive regulation of cell division, fibroblast growth factor receptor binding, histone H3-K27 trimethylation, protein kinase A signaling, and beta-catenin-TCF complex assembly (**Table S8**). Subsequently, we performed multivariate survival analysis on these DEGs in the R platform, and we identified 29 DEGs that are significantly related to the OS of COAD (**Table S9, Figure 10A**). The top three significance DEGs were cytochrome C oxidase subunit 8C (COX8C, log-rank P < 0.0001, adjusted P = 0.003, HR = 1.894, 95%CI = 1.242-2.887, **Figure 10B**), keratin 3 (KRT3, log-rank P = 0.0052, adjusted P = 0.003, HR = 1.890, 95%CI = 1.234-2.896, **Figure 10C**) and collectin subfamily member 10 (COLEC10, log-rank P = 0.0085, adjusted P = 0.008, HR = 0.561, 95%CI = 0.367-0.858, **Figure 10D**). We also used these DEGs to predict STXBP5-AS1 targeted small molecule drugs in the CMap online tool, and we obtained a total of four potential STXBP5-AS1 targeted therapeutic drugs. These four small molecule drugs are anisomycin, cephaeline, NU-1025 and quipazine. The CMap results and the chemical structures of these four small molecule drugs are shown in **Figure 11A-E**. Drug-gene interaction analysis through STITCH suggests that some of the gene targets that interact with these four small molecule drugs are DEGs between the high- and low-STXBP5-AS1 phenotypes (**Figure 12**).

STITCH analysis suggests that anisomycin may target STXBP5-AS1 by acting on HSPB3 and DUSP9, while cephaeline may interact with SERPIN9. STITCH analysis also found that there are many DEGs that interact with quipazine, including HTR3C, CHRNB2, CHRNA9, CHRNA4, CHRNA2, SLC6A15, SLC6A3, CHRNA7, CHRND, HTR3E. We have not observed an interaction between NU-1025 and DEGs between the high- and low-STXBP5-AS1 phenotypes.

**Figure 13:**
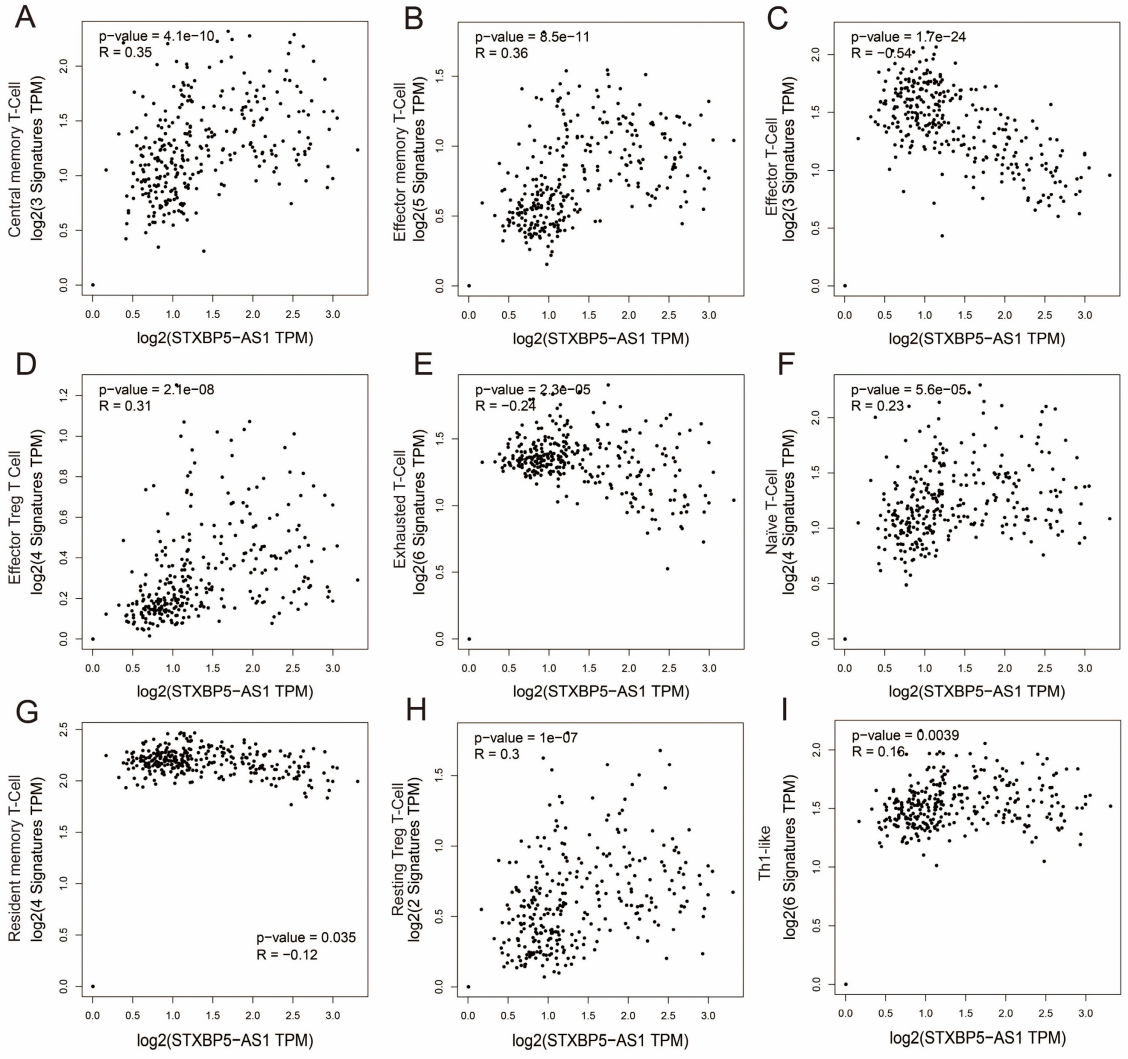
Scatter plot of co-expression correlation between STXBP5-AS1 and nine immune cell gene sets in normal intestinal tissues. (A) Central memory T-Cell; (B) Effector memory T-Cell; (C) Effector T-Cell; (D) Effector Treg T-Cell; (E) Exhausted T-Cell; (F) Naïve T-Cell; (G) Resident memory T-Cell; (H) Resting Treg T-Cell; (I) Th1-like.

**Figure 14:**
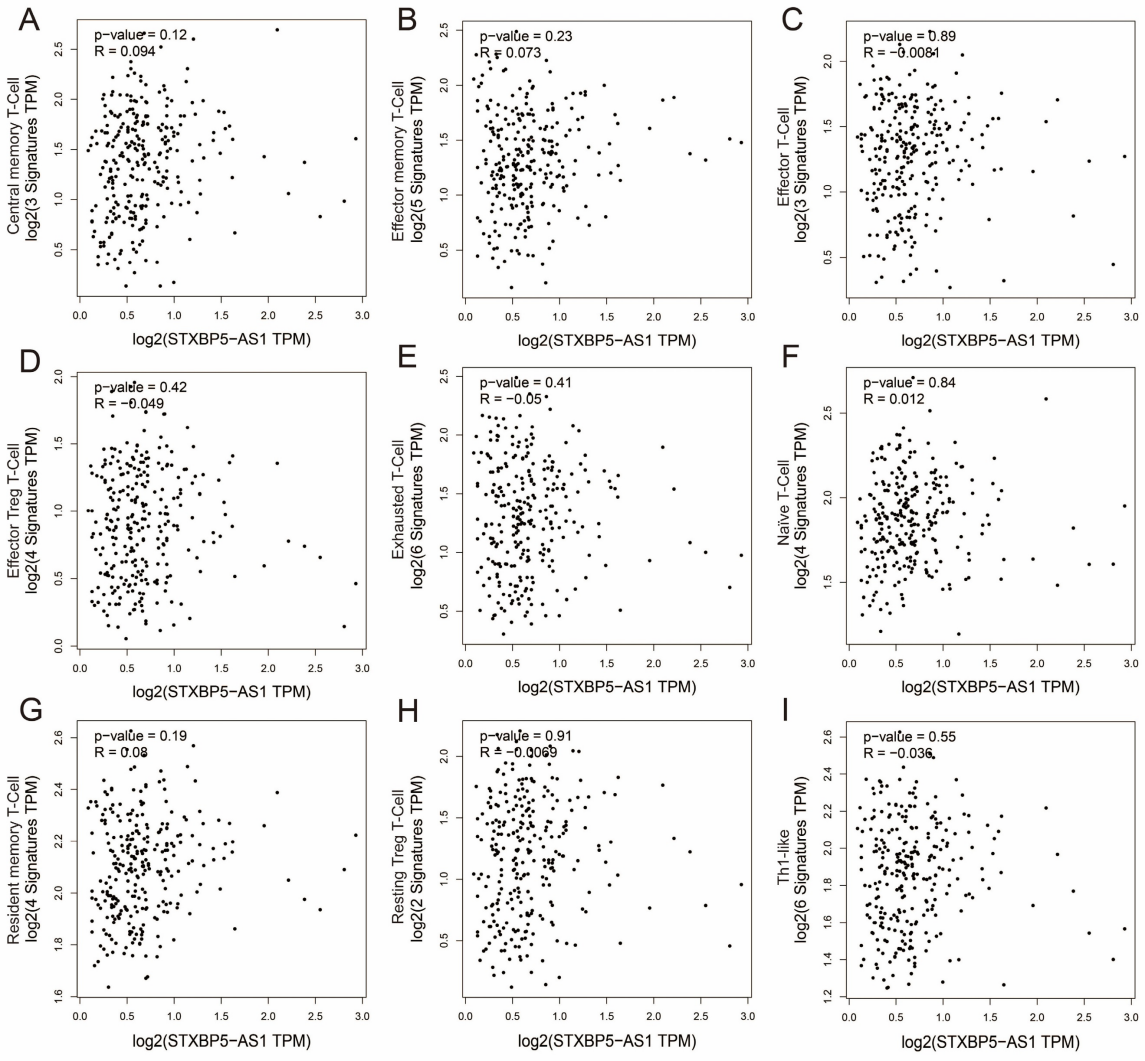
Scatter plot of co-expression correlation between STXBP5-AS1 and nine immune cell gene sets in COAD tumor tissues. (A) Central memory T-Cell; (B) Effector memory T-Cell; (C) Effector T-Cell; (D) Effector Treg T-Cell; (E) Exhausted T-Cell; (F) Naïve T-Cell; (G) Resident memory T-Cell; (H) Resting Treg T-Cell; (I) Th1-like.

We all know that the immune function of the human body is closely related to the occurrence and development of tumors. To further understand the relationship between STXBP5-AS1 and immune cell infiltration, we used GEPIA2 to investigate the relationship between STXBP5-AS1 and immune cell gene sets, which including naïve T-cell, effector T-cell, effector memory T-cell, central memory T-cell, resident memory T-cell, exhausted T-cell, resting Treg T-cell, effector Treg T-cell and Th1-like (**Table S10**). Co-expression analysis found that STXBP5-AS1 showed a significant co-expression correlation with the nine immune cell gene sets in normal intestinal tissues, which was negatively correlated with the effector T-cell and exhausted T-cell gene sets, and with other seven immune cell gene sets are positively correlated (**Figure 13A-I**). In COAD tumor tissues, we did not observe a significant co-expression relationship between STXBP5-AS1 and the above-mentioned nine immune cell gene sets (**Figure 14A-I**). The above analysis results suggest that STXBP5-AS1 has immune disorders in COAD. In normal intestinal tissues, STXBP5-AS1 is controlled by immune cells. Therefore, the expression of STXBP5-AS1 is significantly related to immune cell gene set, while in COAD tumor tissues, STXBP5-AS1 is not controlled by immune cells, so we observed that STXBP5-AS1 is not related to immune cell gene sets in COAD tumor tissues.

## Discussion

Previous studies have shown that STXBP5-AS1 plays a role as tumor suppressor genes in multiple cancers, and is closely related to tumor progression, prognosis and radiotherapy sensitivity. Chen et al. conducted RNA sequencing on lung cancer cell lines after ionizing radiation intervention and found that STXBP5-AS1 was differentially expressed in radiotherapy intervention cell lines. Based on this, they speculated that it may be involved in the regulation of lung cancer radiotherapy sensitivity 20. Ham et al. found that Ginsenoside Rg3 and Korean Red Ginseng extract epigenetically can participate in the regulation of the proliferation and apoptosis of cancer cells by participating in the regulation of STXBP5-AS1 expression in MCF-7 cell lines 21. Shao et al. found that STXBP5-AS1 is significantly low expression in cervical cancer (CC), and the prognosis of CC patients with low STXBP5-AS1 expression is poor. Overexpression of STXBP5-AS1 in the CC cell line can significantly inhibit the proliferation and invasion of tumor cells 5. Cen et al. changed the expression of STXBP5-AS1 in gastric cancer cell lines and found that the proliferation, migration and invasion of cell lines with different expressions of STXBP5-AS1 were affected. In addition, downstream signaling pathway investigation also revealed that the mechanism of STXBP5-AS1 in gastric cancer is achieved by regulating phosphatidylinositol 3 kinase/protein kinase B (PI3K/AKT) signaling pathway 4. Huang et al. found that STXBP5-AS1 is significantly down-regulated in non-small-cell lung carcinoma (NSCLC) tumor tissues, and its expression level is closely related to NSCLC metastasis. *In vitro* experiments confirmed that up-regulating the expression level of STXBP5-AS1 in lung cancer cell lines can significantly inhibit the proliferation, migration and invasion of cancer cells. The downstream molecular mechanism investigation found that STXBP5-AS1 functions through the PI3K/AKT signaling pathway in NSCLC 6. Guo et al. conducted survival analysis through the TCGA breast cancer cohort and found that STXBP5-AS1 is significantly related to the prognosis of breast cancer, and high expression of STXBP5-AS1 indicates an unfavourable prognosis 22. In conclusion, we found that STXBP5-AS1 is down-regulated in tumor tissues in previous studies and low STXBP5-AS1 expression indicates poor prognosis of cancers, except for breast cancer. Functional analysis suggests that STXBP5-AS1 plays a tumor suppressor role in cancers. The results of our currnet study also consistent with the above studies by using TGCA COAD cohort dataset. We also found that STXBP5-AS1 is significantly lower expression in COAD tumor tissues. Survival analysis suggests that patients with low expression of STXBP5-AS1 have a shorter OS.

In the functional enrichment of STXBP5-AS1 co-expressed PCGs, GSEA and DEGs between low- and high-STXBP5-AS1 phenotypes, we have initially identified multiple biological functions and pathways that may be the molecular mechanism of STXBP5-AS1 in COAD. As a classic cancer-related pathway, the Wnt signaling pathway is closely related to tumor stem cells, microenvironment, invasion, proliferation and other tumor biological behaviors of colorectal cancer. It can also be used as a potential target for colorectal cancer targeted therapy 23-25. Li et al. analyzed TCGA pan-cancer data and found that TP53 mutation is closely related to tumor immunity in multiple cancers, and TP53 mutation is a potential prognostic marker for multiple cancers, including colorectal cancer 26. Previous studies found that NOTCH4 was significantly highly expressed in colorectal cancer tissues, especially in distant metastatic colorectal cancer tissues, and survival analysis suggested that NOTCH4 could be used as a prognostic marker for colorectal cancer, while patients with high expression of NOTCH4 had a poor prognosis 27, 28. *In vitro* functional experiments suggested that changing the expression level of NOTCH4 in colorectal cancer could significantly affect the proliferation and invasion of colorectal cancer cells 29. Wang et al. also showed that patients with positive immunohistochemical expression of NOTCH4 had a poor prognosis, and its expression was also closely related to lymph node invasion and metastasis of breast cancer 30. Myc can be used as a transcription regulator of lymphoid enhancer factor 1 to regulate the proliferation of colon cancer cells, and its molecular mechanism is realized by regulating the downstream Wnt signaling pathway 31. In addition, other study also showed that kynurenine pathway could change the uptake and metabolism of tryptophan in colon cancer, thereby affecting the proliferation of colorectal cancer cells 32. Mammalian target of rapamycin complex 1 (mTORC1) is widely reported to be closely related to tumor associated blood vessels and tumor immunity, it has also been reported to act as a gateway for autophagy 33, 34. Fricke et al. also reported that mTORC1 inhibitors could serve as therapeutic targets for PIK3CA mutant colorectal cancer 35. Previous studies have suggested that the cGMP-PKG signaling pathway is closely related to the treatment of colorectal cancer and the proliferation of cancer cells 36. cGMP-PKG signaling pathway activated by nitric oxide can significantly affect the migration and invasion of colorectal cancer tumor cells 37. Sulindac can selectively inhibit the proliferation of colorectal cancer cells by activating the cGMP-PKG signaling pathway through Wnt/b-Catenin signaling pathway 38. Through the above literature review of some of the biological processes and pathways enriched in this study, we found that a large number of molecular mechanisms of STXBP5-AS1 have been reported to be closely related to colorectal cancer.

For the four drugs identified in the present study, we found that there were no previous studies had reported the roles of cephaeline and quipazine in cancers. As a poly (ADP-Ribose) polymerase (PARP) inhibitor, NU-1025, can regulate Tp53 in human glioblastoma cells, thereby affecting the sensitivity of tumor cells to radiotherapy 39. Wesierska-gadek et al. found that NU-1025 showed strong cytotoxicity in BRCA1-positive breast cancer cell lines, but no effect in BRCA1-negative breast cancer cell lines 40. Liu et al. identified NU-1025 as a potential treatment drug by bioinformatics methods, in addition, NU-1025 has also been confirmed to play a neuroprotective effect in Cerebral Ischemia 41, 42. Anisomycin has been widely reported in the previous studies and is closely related to cancers. Anisomycin has been reported to have significant tumor suppressor effect in colorectal cancer. Intervention with anisomycin in cell lines can significantly block the cell cycle, proliferation and induce apoptosis of cancer cells 43, 44. Anisomycin also has been reported to have a suppressive effect on ovarian cancer, and can significantly inhibiting angiogenesis, proliferation, and invasion of cancer cells 45, 46. Anisomycin also exerts an anti-cancer effect in osteosarcoma, and can enhance the anti-tumor effect of doxorubicin 47. Anisomycin has also been reported to be related to tumor immunity in cancers, Lee et al. found that anisomycin can mediate natural killer cells to exert anti-cancer effects in hepatocellular carcinoma (HCC) 48. In addition, study have shown that anisomycin can participate in inducing HCC cell apoptosis through mediating mitochondrial related pathways 49. Anisomycin can inhibit the proliferation of chronic myeloid leukemia (CML) CD34 stem cells and induce apoptosis, and the combination of imatinib or dasatinib can significantly improve their efficacy. Through this study, the authors believe that anisomycin can be used to treat BCR-ABL tyrosine kinase inhibitors resistant CML patients 50. Anisomycin can mediate the apoptosis of tumor cells through the death receptor 4 gene. In addition, anisomycin combined with mapatumumab treatment has a coordinated effect, which can significantly improve the cytotoxicity to renal cell carcinoma 51. Anisomycin has also been reported to induce cancer cell apoptosis in breast cancer 52, 53. Anisomycin can also induce the apoptosis of glucocorticoid resistant lymphoblastic cells through the p38 /JUN pathway 54, 55.

Anisomycin induces apoptosis in cancers by a diversified mechanism, which can induce apoptosis in a variety of cancers, including kidney cancer, breast cancer and human glioma, by regulating the bcl-2, C-flip (L) and McL-1 pathways 56. Slipicevic et al. reported that that anisomycin combined with lexatumumab can synergistically induce apoptosis of melanoma cells 57. Anisomycin induces cancer cell death in human glioblastoma cell lines in a time- and concentration-dependent manner, and its mechanism of action is through down-regulation of the PP2A catalytic subunit 58. Anisomycin in malignant mesothelioma cells can induce apoptosis by increasing the sensitivity of tumor cells to tumor necrosis factor-related apoptosis inducing ligand 59. As an effective JNK agonist, anisomycin plays a role in prostate carcinoma cells through Fas-mediated apoptosis induced by JNK 60. In addition, anisomycin in AML cell line HL-60 also induces tumor cell apoptosis through JNK/SAPK pathway 61. Through literature review, we found that among the four COAD targeted drugs identified in the present study, anisomycin and NU-1025 are widely reported as tumor suppressor drugs. The above results indicate that our drug screening strategy is reliable. In addition, we were also identified for the first time that cephaeline and quipazine can be used as a targeted therapy drugs for COAD.

This study has certain limitations. First, this study is a single-cohort study and lacks independent cohort verification. Secondly, this study is an *in silico* study based on whole-genome RNA sequencing dataset, and lacks* in vivo* and *in vitro* experimental verification. Third, the clinical parameters obtained from TCGA in this study is not complete, and the multivariate survival analysis model failed to include some well-known COAD prognostic-related clinical parameters for adjustment. Despite the above limitations, this study is the first to report STXBP5-AS1 prognostic value in COAD, and to fully investigate the molecular mechanisms and targeted drugs of STXBP5-AS1 in COAD by using the whole genome RNA sequencing dataset. This study provides original innovation and source innovations on the prognostic value and molecular mechanism of STXBP5-AS1 in COAD. This study preliminarily screened and identified the molecular mechanism of STXBP5-AS1 in COAD, which could provide theoretical basis and research direction for future studies of STXBP5-AS1. Once the above-mentioned mechanism of action and prognostic value are verified in large cohorts and *in vivo* and *in vitro* experiments, it can provide new strategies for the treatment of COAD.

## Conclusions

In conclusion, our study for the first time found that STXBP5-AS1 was significantly down-regulated in COAD tumor tissues, and low expression of STXBP5-AS1 predicted poor OS of COAD. Functional enrichment analysis found that STXBP5-AS1 may be involved in cell junction, DNA replication, apoptosis, cell cycle, metastasis, TP53, Wnt, mTORC1, MCM, NOTCH4, transforming growth factor beta receptor, and cGMP-PKG signaling pathways and biological processes in COAD. At the same time, we also preliminarily identified four small-molecule targeted therapy drugs for STXBP5-AS1 using CMap. As this study still has certain shortcomings, however, our results still need to be confirmed in future studies.

## Supplementary Material

Supplementary tables.

## Figures and Tables

**Table 1:** Joint effects survival analysis of tumor stage and STXBP5-AS1 expression in COAD OS.

Group	STXBP5-AS1	Tumor stage^†^	Patients (n=438)	MST (days)	Crude HR (95% CI)	Crude *P*	Adjusted HR (95% CI)	Adjusted *P* £
								
A	**High expression**	**I**	38	NA	1		1	
B	**High expression**	**II**	81	NA	1.282(0.357-4.611)	0.703	1.282(0.357-4.611)	0.703
C	**High expression**	**III**	63	NA	2.404(0.670-8.624)	0.178	2.404(0.670-8.624)	0.178
D	**High expression**	**IV**	33	2003	4.192(1.167-15.060)	0.028	4.192(1.167-15.060)	0.028
E	**Low expression**	**I**	35	NA	0.370(0.038-3.562)	0.390	0.370(0.038-3.562)	0.390
F	**Low expression**	**II**	86	2821	1.836(0.534-6.312)	0.335	1.836(0.534-6.312)	0.335
G	**Low expression**	**III**	63	NA	3.177(0.941-10.726)	0.063	3.177(0.941-10.726)	0.063
H	**Low expression**	**IV**	28	334	15.464(4.581-52.203)	<0.0001	15.464(4.581-52.203)	<0.0001
								
I	**High expression**	**I+II**	119	NA	1		1	
II	**High expression**	**III+IV**	96	2003	2.466(1.257-4.838)	0.009	83656(2.760-27.150)	<0.0001
III	**Low expression**	**I+II**	121	3042	1.221(0.601-2.478)	0.581	1.210(0.596-2.456)	0.599
IV	**Low expression**	**III+IV**	91	1331	4.368(2.374-8.037)	<0.0001	19.893(6.398-61.850)	<0.0001

**Notes**: £Adjusted for histological grade, radiation therapy, radical resection and targeted molecular therapy. † Tumor stage information are unavailable in 11 patients. **Abbreviation:** STXBP5-AS1, STXBP5 Antisense RNA 1; OS, overall survival; COAD, colon adenocarcinoma; MST, median survival time; HR, hazard ratio; CI, confidence interval.
