# Supplementary material for: Comprehensive investigation of the prognostic values and molecular mechanisms of syntaxin binding protein 5 antisense RNA 1 in patients with colon adenocarcinoma based on RNA sequencing dataset
Source: J Cancer. 2023 Jun 4;14(9):1607–22. doi: 10.7150/jca.83423 (PMC10266242; doi:10.7150/jca.83423)
Supplement: Supplementary file 1 — Supplementary tables. [file jcav14p1607s1.pdf]

Table S1. Clinical parameters of TCGA COAD cohort

Variables	Patients (n=438)	MST(days)	HR(85%CI)	Log-rank P
**Gender**				0.545
**Female**	204	NA	1	
**Male**	234	2475	1.131(0.759-1.686)	
**Age (years)§**				0.112
**<65**	168	NA	1	
**≥65**	268	2475	1.420(0.919-2.194)	
**Tumor stage £**				<0.0001
**I**	73	NA	1	
**II**	167	2821	2.240(0.781-6.421)	
**III**	126	NA	4.068(1.434-11.538)	
**IV**	61	858	11.291(3.980-32.026)	
**Tumor stage**				<0.0001
**I+II**	240	3042	1	
**III+IV**	187	1711	3.097(2.009-4.772)	
**STXBP5AS1**				0.035
**Low expression**	219	2821	1	
**High expression**	219	2532	0.648(0.431-0.973)	

Notes: §Information of age was unavailable in 2 patients; £Information of tumor stage was unavailable in 11 patients.

Table S2. Co-expressed proteins encoding genes list of STXBP5-AS1 in COAD tumor tissues (the correlation coefficient ranked top20 up-regulated genes and down-regulated genes).

ID	ID	R	P Value
**STXBP5-AS1**	BIN1	-0.302	0.009133
**STXBP5-AS1**	PSMC4	-0.29	0.01205
**STXBP5-AS1**	VAV2	-0.286	0.013191
**STXBP5-AS1**	GNPDA1	-0.285	0.013491
**STXBP5-AS1**	POLDIP2	-0.281	0.014752
**STXBP5-AS1**	RUVBL1	-0.276	0.016472
**STXBP5-AS1**	AIFM2	-0.275	0.016837
**STXBP5-AS1**	SAE1	-0.274	0.017209
**STXBP5-AS1**	SLC25A39	-0.273	0.017588
**STXBP5-AS1**	LRRC20	-0.27	0.018768
**STXBP5-AS1**	COPRS	-0.269	0.019177
**STXBP5-AS1**	TPD52L2	-0.266	0.02045
**STXBP5-AS1**	BCL2L12	-0.266	0.02045
**STXBP5-AS1**	C10orf35	-0.264	0.021339
**STXBP5-AS1**	SMYD5	-0.259	0.023711
**STXBP5-AS1**	ATIC	-0.257	0.024722
**STXBP5-AS1**	CCT7	-0.256	0.025241
**STXBP5-AS1**	CHCHD6	-0.256	0.025241
**STXBP5-AS1**	IFITM3	-0.255	0.02577
**STXBP5-AS1**	GRINA	-0.255	0.02577
**STXBP5-AS1**	TMEM210	0.542	3.05E-05
**STXBP5-AS1**	RAP1GAP	0.546	2.65E-05
**STXBP5-AS1**	CCDC60	0.552	2.14E-05
**STXBP5-AS1**	GNE	0.552	2.14E-05
**STXBP5-AS1**	TCN1	0.562	1.49E-05
**STXBP5-AS1**	STXBP5	0.563	1.44E-05
**STXBP5-AS1**	FAM174B	0.57	1.11E-05
**STXBP5-AS1**	ARFGEF3	0.577	8.56E-06
**STXBP5-AS1**	CAPN9	0.577	8.56E-06
**STXBP5-AS1**	NEURL1	0.578	8.25E-06
**STXBP5-AS1**	LRRIQ4	0.584	6.58E-06
**STXBP5-AS1**	HEPACAM2	0.584	6.58E-06
**STXBP5-AS1**	ADAM2	0.585	6.34E-06
**STXBP5-AS1**	PLD1	0.609	2.51E-06
**STXBP5-AS1**	RHBDL3	0.614	2.06E-06
**STXBP5-AS1**	ANO7	0.636	8.47E-07
**STXBP5-AS1**	B3GNT6	0.653	4.18E-07
**STXBP5-AS1**	SMIM14	0.66	3.10E-07
**STXBP5-AS1**	GALNT8	0.668	2.20E-07
**STXBP5-AS1**	REP15	0.691	8.06E-08

Table S3. Function enrichment results of STXBP5-AS1 co-expressed genes.

Category	Term	Count	PValue
**KEGG_PATHWAY**	hsa00512:Mucin type O-Glycan biosynthesis	10	1.17E-06
**KEGG_PATHWAY**	hsa04141:Protein processing in endoplasmic reticulum	18	1.69E-04
**KEGG_PATHWAY**	hsa05014:Amyotrophic lateral sclerosis (ALS)	7	0.010082
**KEGG_PATHWAY**	hsa04922:Glucagon signaling pathway	10	0.011098
**KEGG_PATHWAY**	hsa04024:cAMP signaling pathway	15	0.015622
**KEGG_PATHWAY**	hsa04144:Endocytosis	17	0.017592
**KEGG_PATHWAY**	hsa04924:Renin secretion	7	0.030995
**KEGG_PATHWAY**	hsa04022:cGMP-PKG signaling pathway	12	0.033195
**KEGG_PATHWAY**	hsa05031:Amphetamine addiction	7	0.035347
**GOTERM_CC_DIRECT**	GO:0000139~Golgi membrane	57	6.72E-10
**GOTERM_CC_DIRECT**	GO:0016020~membrane	136	3.05E-08
**GOTERM_CC_DIRECT**	GO:0005794~Golgi apparatus	66	2.43E-07
**GOTERM_MF_DIRECT**	GO:0004653~polypeptide N-acetylgalactosaminyltransferase activity	8	7.56E-06
**GOTERM_BP_DIRECT**	GO:0006468~protein phosphorylation	37	6.09E-05
**GOTERM_MF_DIRECT**	GO:0005515~protein binding	399	7.91E-05
**GOTERM_CC_DIRECT**	GO:0005829~cytosol	166	2.81E-04
**GOTERM_BP_DIRECT**	GO:0043547~positive regulation of GTPase activity	40	4.92E-04
**GOTERM_BP_DIRECT**	GO:0016266~O-glycan processing	10	5.27E-04
**GOTERM_CC_DIRECT**	GO:0005783~endoplasmic reticulum	51	0.001341
**GOTERM_BP_DIRECT**	GO:0006486~protein glycosylation	13	0.001641
**GOTERM_MF_DIRECT**	GO:0005524~ATP binding	83	0.00175
**GOTERM_BP_DIRECT**	GO:0006047~UDP-N-acetylglucosamine metabolic process	4	0.001887
**GOTERM_CC_DIRECT**	GO:0070382~exocytic vesicle	5	0.002188
**GOTERM_BP_DIRECT**	GO:0051056~regulation of small GTPase mediated signal transduction	14	0.002429
**GOTERM_MF_DIRECT**	GO:0005085~guanyl-nucleotide exchange factor activity	13	0.002602
**GOTERM_MF_DIRECT**	GO:0017137~Rab GTPase binding	14	0.002676
**GOTERM_MF_DIRECT**	GO:0004571~mannosyl-oligosaccharide 1,2-alpha-mannosidase activity	4	0.003027
**GOTERM_MF_DIRECT**	GO:0005089~Rho guanyl-nucleotide exchange factor activity	10	0.003428
**GOTERM_BP_DIRECT**	GO:0030193~regulation of blood coagulation	5	0.003759
**GOTERM_BP_DIRECT**	GO:0043065~positive regulation of apoptotic process	23	0.003909
**GOTERM_BP_DIRECT**	GO:0035023~regulation of Rho protein signal transduction	10	0.004481
**GOTERM_MF_DIRECT**	GO:0030246~carbohydrate binding	17	0.005274
**GOTERM_BP_DIRECT**	GO:0006895~Golgi to endosome transport	5	0.005751
**GOTERM_BP_DIRECT**	GO:0035265~organ growth	4	0.005922

**GOTERM_CC_DIRECT**	GO:0005737~cytoplasm	234	0.006491
**GOTERM_CC_DIRECT**	GO:0042589~zymogen granule membrane	4	0.007626
**GOTERM_CC_DIRECT**	GO:0030667~secretory granule membrane	5	0.007976
**GOTERM_BP_DIRECT**	GO:0045921~positive regulation of exocytosis	5	0.008344
**GOTERM_BP_DIRECT**	GO:0051402~neuron apoptotic process	6	0.008857
**GOTERM_BP_DIRECT**	GO:0034976~response to endoplasmic reticulum stress	9	0.009159
**GOTERM_BP_DIRECT**	GO:0048208~COPII vesicle coating	8	0.0098
**GOTERM_BP_DIRECT**	GO:0007399~nervous system development	21	0.009877
**GOTERM_BP_DIRECT**	GO:0045332~phospholipid translocation	5	0.009887
**GOTERM_MF_DIRECT**	GO:0005351~sugar:proton symporter activity	4	0.010556
**GOTERM_BP_DIRECT**	GO:0006888~ER to Golgi vesicle-mediated transport	14	0.010705
**GOTERM_CC_DIRECT**	GO:0043231~intracellular membrane-bounded organelle	34	0.011141
**GOTERM_CC_DIRECT**	GO:0005789~endoplasmic reticulum membrane	48	0.011974
**GOTERM_BP_DIRECT**	GO:0006886~intracellular protein transport	18	0.012332
**GOTERM_BP_DIRECT**	GO:0043087~regulation of GTPase activity	8	0.013679
**GOTERM_CC_DIRECT**	GO:0005811~lipid particle	8	0.013868
**GOTERM_MF_DIRECT**	GO:0030742~GTP-dependent protein binding	5	0.01402
**GOTERM_MF_DIRECT**	GO:0016740~transferase activity	10	0.014209
**GOTERM_BP_DIRECT**	GO:0045053~protein retention in Golgi apparatus	3	0.014293
**GOTERM_MF_DIRECT**	GO:0030618~transforming growth factor beta receptor, pathway-specific cytoplasmic mediator activity	3	0.014605
**GOTERM_MF_DIRECT**	GO:0016887~ATPase activity	15	0.014847
**GOTERM_BP_DIRECT**	GO:0007257~activation of JUN kinase activity	6	0.015956
**GOTERM_MF_DIRECT**	GO:0005096~GTPase activator activity	20	0.016462
**GOTERM_BP_DIRECT**	GO:0060527~prostate epithelial cord arborization involved in prostate glandular acinus morphogenesis	3	0.020885
**GOTERM_BP_DIRECT**	GO:1903799~negative regulation of production of miRNAs involved in gene silencing by miRNA	3	0.020885
**GOTERM_BP_DIRECT**	GO:0060348~bone development	6	0.023801
**GOTERM_BP_DIRECT**	GO:0009791~post-embryonic development	8	0.024522
**GOTERM_CC_DIRECT**	GO:0030173~integral component of Golgi membrane	7	0.024596
**GOTERM_BP_DIRECT**	GO:0007030~Golgi organization	8	0.0262
**GOTERM_MF_DIRECT**	GO:0046332~SMAD binding	6	0.027218
**GOTERM_MF_DIRECT**	GO:0005544~calcium-dependent phospholipid binding	7	0.027286
**GOTERM_CC_DIRECT**	GO:0014069~postsynaptic density	14	0.027686
**GOTERM_MF_DIRECT**	GO:0004674~protein serine/threonine kinase activity	24	0.027891
**GOTERM_MF_DIRECT**	GO:0019902~phosphatase binding	6	0.032398
**GOTERM_CC_DIRECT**	GO:0043195~terminal bouton	7	0.032852

**GOTERM_BP_DIRECT**	GO:0060395~SMAD protein signal transduction	7	0.034681
**GOTERM_CC_DIRECT**	GO:0030054~cell junction	27	0.034955
**GOTERM_BP_DIRECT**	GO:0009306~protein secretion	5	0.035723
**GOTERM_CC_DIRECT**	GO:0071141~SMAD protein complex	3	0.036118
**GOTERM_CC_DIRECT**	GO:0045211~postsynaptic membrane	15	0.03637
**GOTERM_BP_DIRECT**	GO:0050767~regulation of neurogenesis	4	0.036781
**GOTERM_BP_DIRECT**	GO:0060314~regulation of ryanodine-sensitive calcium-release channel activity	4	0.036781
**GOTERM_BP_DIRECT**	GO:0006623~protein targeting to vacuole	3	0.037001
**GOTERM_BP_DIRECT**	GO:0051963~regulation of synapse assembly	3	0.037001
**GOTERM_BP_DIRECT**	GO:0072659~protein localization to plasma membrane	7	0.037108
**GOTERM_MF_DIRECT**	GO:0043023~ribosomal large subunit binding	3	0.037774
**GOTERM_BP_DIRECT**	GO:0070059~intrinsic apoptotic signaling pathway in response to endoplasmic reticulum stress	5	0.039429
**GOTERM_BP_DIRECT**	GO:0002931~response to ischemia	5	0.039429
**GOTERM_CC_DIRECT**	GO:0016529~sarcoplasmic reticulum	5	0.041637
**GOTERM_BP_DIRECT**	GO:0006491~N-glycan processing	4	0.042042
**GOTERM_CC_DIRECT**	GO:0016023~cytoplasmic, membrane-bounded vesicle	11	0.042886
**GOTERM_MF_DIRECT**	GO:0005509~calcium ion binding	39	0.045884
**GOTERM_BP_DIRECT**	GO:0032330~regulation of chondrocyte differentiation	3	0.046353
**GOTERM_MF_DIRECT**	GO:0047144~2-acylglycerol-3-phosphate O-acyltransferase activity	3	0.047308
**GOTERM_MF_DIRECT**	GO:0030145~manganese ion binding	6	0.047887

Table S4. Survival analysis results of STXBP5-AS1 co-expressed genes in COAD (Only gene list with P<0.05 is shown).

ID	P	HR	Low 95%CI	High 95%CI
**EVI5**	0.002106	0.502696	0.324275	0.779286
**ABCA5**	0.002836	0.523879	0.342671	0.800911
**CCNJL**	0.005046	0.548641	0.360626	0.834678
**ZBTB7C**	0.005864	0.551929	0.361638	0.842349
**LRBA**	0.007459	0.560966	0.367303	0.856738
**FAM167A**	0.007667	0.563147	0.369243	0.858877
**LPCAT2**	0.00824	0.560875	0.365231	0.86132
**COTL1**	0.00898	1.747004	1.149586	2.654887
**BMPR1B**	0.009567	1.74226	1.144804	2.65152
**GALNT7**	0.009695	0.56936	0.37156	0.872459
**USP53**	0.010274	0.577029	0.379165	0.878147
**APAF1**	0.010884	0.574624	0.375129	0.880211
**PSME3**	0.011093	0.583492	0.385016	0.88428
**KIAA0319L**	0.011404	0.582529	0.383281	0.885356
**RALGPS2**	0.011436	0.584981	0.386086	0.886337
**SHC2**	0.012012	1.70986	1.125071	2.598609
**AFF1**	0.012454	0.588305	0.388063	0.891873
**CLCA1**	0.013016	0.581445	0.379008	0.892009
**B3GNT6**	0.013865	0.586306	0.383209	0.897041
**DSG2**	0.014211	0.592215	0.389589	0.900228
**EDEM3**	0.01608	0.601405	0.397516	0.90987
**TYSND1**	0.017058	1.662661	1.094924	2.524779
**GIF**	0.01848	0.603991	0.397062	0.918762
**TPD52L1**	0.02326	1.627088	1.068575	2.477519
**CD81**	0.024003	1.611978	1.06487	2.44018
**REP15**	0.029968	0.62727	0.411677	0.955768
**GCC2**	0.030666	0.631947	0.416808	0.958133
**CCDC62**	0.031351	0.632782	0.41715	0.959876
**MFSD6**	0.032487	0.631068	0.413839	0.962323
**DENND1B**	0.035033	0.641474	0.42452	0.969306
**SLC35B3**	0.035618	0.639652	0.421636	0.970399
**ADGRL3**	0.036186	0.640228	0.42183	0.971699
**KIAA1147**	0.038056	0.641952	0.422291	0.975873
**SCAMP1**	0.038236	0.646226	0.427611	0.976608
**NFS1**	0.038725	0.645601	0.426362	0.977576
**PCSK1**	0.038742	0.645176	0.425799	0.977578
**CHD9**	0.04042	0.645762	0.425046	0.981089
**SEC24A**	0.041295	0.648329	0.427586	0.983031
**C10orf35**	0.041553	1.539204	1.016645	2.330361
**TSPAN13**	0.042475	1.53412	1.014634	2.319579
**STXBP5**	0.047552	0.652801	0.428116	0.995407
**CREB3L1**	0.047625	1.524007	1.00442	2.312377

Table S5. GSEA analysis between low- and high-STXBP5-AS1 phenotype using the c2 reference gene set.

NAME	SIZE	ES	NES	NOM p-val	FDR q-val
HESS_TARGETS_OF_HOXA9_AND_MEIS1_UP	66	0.544516	1.806434	0.008247	0.127982
NADERI_BREAST_CANCER_PROGNOSIS_UP	50	0.57856	1.807527	0.016327	0.128577
REACTOME_MRNA_CAPPING	29	0.614067	1.818318	0.002079	0.128598
REACTOME_ASYMMETRIC_LOCALIZATION_OF_PCP_PROTEINS	63	0.585912	1.808923	0.01875	0.128802
REACTOME_STABILIZATION_OF_P53	55	0.636658	1.794782	0.028692	0.129698
REACTOME_G1_S_DNA_DAMAGE_CHECKPOINTS	66	0.610456	1.80941	0.014768	0.130037
REACTOME_DNA_REPLICATION_PRE_INITIATION	84	0.65456	1.818806	0.012579	0.130039
REACTOME_RRNA_PROCESSING_IN_THE_NUCLEUS_AND_CYTOSOL	189	0.638116	1.823347	0.018367	0.13018
KEGG_HOMOLOGOUS_RECOMBINATION	28	0.635265	1.793106	0.011719	0.130211
REACTOME_HEDGEHOG_OFF_STATE	111	0.463466	1.819778	0.014344	0.130746
BASAKI_YBX1_TARGETS_UP	280	0.510387	1.814545	0.025	0.131095
KIM_ALL_DISORDERS_DURATION_CORR_DN	140	0.584349	1.824287	0.020284	0.131098
REACTOME_MITOCHONDRIAL_PROTEIN_IMPORT	60	0.674115	1.79484	0.012448	0.131255

MATTIOLI_MGUS_VS_PCL	9	0.4	1.8	0.00	0.1
	9	999	113	833	312
		03	82	3	85
REACTOME_DEGRADATION_OF_DVL	5	0.6	1.8	0.01	0.1
	6	425	206	242	316
		23	39	2	3
SCHLOSSER_MYC_TARGETS_AND_SERUM_RESPONSE_UP	4	0.6	1.7	0.00	0.1
	6	033	973	806	316
		48	94	5	58
REACTOME_RNA_POLYMERASE_II_TRANSCRIPTION_ELONGATI	5	0.5	1.8	0.00	0.1
ON	7	280	095	404	317
		03	63		68
REACTOME_FBXL7_DOWN_REGULATES_AURKA_DURING_MIT	5	0.6	1.7	0.01	0.1
OTIC_ENTRY_AND_IN_EARLY_MITOSIS	4	641	984	659	321
		46	14	8	34
REACTOME_DNA_REPLICATION	1	0.6	1.8	0.00	0.1
	2	209	123	833	321
	6	89	21	3	63
GINESTIER_BREAST_CANCER_ZNF217_AMPLIFIED_DN	3	0.5	1.8	0.01	0.1
	1	836	250	571	321
	6	81	82	7	68
REACTOME_DEGRADATION_OF_AXIN	5	0.6	1.7	0.02	0.1
	4	268	951	249	326
		05	06	5	11
GRADE_COLON_AND_RECTAL_CANCER_UP	2	0.5	1.7	0.01	0.1
	8	131	896	239	326
	7	53	22	7	52
VECCHI_GASTRIC_CANCER_EARLY_UP	4	0.4	1.7	0.02	0.1
	1	985	448	183	328
	5	89	65	4	66
REACTOME_TRANSCRIPTION_OF_THE_HIV_GENOME	6	0.5	1.7	0.01	0.1
	9	134	455	212	331
		52	86	1	99
LEE_METASTASIS_AND_RNA_PROCESSING_UP	1	0.7	1.7	0.00	0.1
	6	204	989	200	333
		57	51	4	61
RHODES_UNDIFFERENTIATED_CANCER	6	0.6	1.8	0.01	0.1
	8	567	001	452	336
		43	6	3	64
REACTOME_ORC1_REMOVAL_FROM_CHROMATIN	7	0.6	1.7	0.01	0.1
	0	525	873	476	337
		67	42	8	59
REACTOME_REGULATION_OF_MITOTIC_CELL_CYCLE	8	0.6	1.7	0.02	0.1
	5	144	637	096	338
		74	75	4	95
WALLACE_JAK2_TARGETS_UP	2	0.6	1.7	0.01	0.1

	2	518	469	814	339
		37	17	5	31
REACTOME_RNA_POLYMERASE_III_TRANSCRIPTION_INITIATIO	2	0.6	1.8	0.00	0.1
N_FROM_TYPE_1_PROMOTER	8	006	253	614	340
		92	47	8	2
TOMIDA_METASTASIS_UP	2	0.5	1.7	0.01	0.1
	6	863	456	467	342
		37	54	5	5
REACTOME_REGULATION_OF_EXPRESSION_OF_SLITS_AND_RO	1	0.6	1.7	0.03	0.1
BOS	6	028	646	125	343
	9	62	23		74
PRAMOONJAGO_SOX4_TARGETS_DN	4	0.5	1.7	0.01	0.1
	7	589	472	670	347
		16	31	1	35
REACTOME_BUTYRATE_RESPONSE_FACTOR_1_BRF1_BINDS_A	1	0.6	1.7	0.01	0.1
ND_DESTABILIZES_MRNA	7	223	480	024	349
		65	39	6	32
REACTOME_TP53_REGULATES_TRANSCRIPTION_OF_DNA_REPA	6	0.5	1.7	0.01	0.1
IR_GENES	1	139	651	609	349
		14	77	7	57
SCHLOSSER_SERUM_RESPONSE_AUGMENTED_BY_MYC	1	0.5	1.8	0.00	0.1
	0	295	259	613	354
	5	27	73	5	99
REACTOME_RESOLUTION_OF_ABASIC_SITES_AP_SITES	3	0.6	1.7	0.00	0.1
	8	105	548	612	356
		48	83	2	23
REACTOME_INFLUENZA_INFECTION	1	0.5	1.7	0.04	0.1
	5	772	483	8	357
	4	74	81		32
REACTOME_GLOBAL_GENOME_NUCLEOTIDE_EXCISION_REPAI	8	0.5	1.7	0.01	0.1
R_GG_NER	4	164	412	919	357
		99	44		84
REACTOME_PREFOLDIN_MEDIATED_TRANSFER_OF_SUBSTRAT	2	0.6	1.7	0.01	0.1
E_TO_CCT_TRIC	8	230	515	927	359
		41	72	2	39
REACTOME_ACTIVATION_OF_APC_C_AND_APC_C:CDC20_MEDI	7	0.6	1.7	0.01	0.1
ATED_DEGRADATION_OF_MITOTIC_PROTEINS	5	368	654	910	36
		38	61	8	
REACTOME_RNA_POLYMERASE_III_TRANSCRIPTION_TERMINA	2	0.5	1.7	0.01	0.1
TION	3	884	565	414	361
		16	92	1	37
REACTOME_COOPERATION_OF_PREFOLDIN_AND_TRIC_CCT_IN	3	0.5	1.7	0.02	0.1
_ACTIN_AND_TUBULIN_FOLDING	3	993	502	132	362
		19	12	2	25
REACTOME_VIF_MEDIATED_DEGRADATION_OF_APOBEC3G	5	0.6	1.7	0.02	0.1
	3	642	585	484	362

		95	3	5	98
REACTOME_NEGATIVE_REGULATION_OF_NOTCH4_SIGNALING	5	0.6	1.7	0.02	0.1
	4	553	553	074	363
		12	3	7	22
REACTOME_DUAL_INCISION_IN_TC_NER	6	0.5	1.7	0.00	0.1
	5	385	662	630	363
		31	62	3	42
REACTOME_RNA_POLYMERASE_III_TRANSCRIPTION_INITIATIO	2	0.5	1.7	0.00	0.1
N_FROM_TYPE_3_PROMOTER	8	755	672	618	364
		79	71	6	3
REACTOME_PCP_CE_PATHWAY	9	0.5	1.7	0.02	0.1
	1	143	838	306	364
		93	03	1	57
REACTOME_S_PHASE	1	0.5	1.7	0.02	0.1
	6	487	485	697	367
	0	15	63	1	05
XU_RESPONSE_TO_TRETINOIN_AND_NSC682994_DN	1	0.7	1.7	<0.0	0.1
	5	260	571	1	367
		57	24		61
KEGG_DNA_REPLICATION	3	0.6	1.7	0.01	0.1
	6	794	518	237	367
		46	63	1	99
REACTOME_DECTIN_1_MEDIATED_NONCANONICAL_NF_KB_SI	6	0.6	1.7	0.02	0.1
GNALING	1	106	524	725	373
		03	15	4	55
NIKOLSKY_BREAST_CANCER_5P15_AMPLICON	2	0.6	1.7	0.01	0.1
	6	454	587	565	373
		27	25	6	66
YAO_TEMPORAL_RESPONSE_TO_PROGESTERONE_CLUSTER_13	1	0.5	1.7	0.02	0.1
	5	729	387	074	375
	9	35	45	7	01
KEGG_PYRIMIDINE_METABOLISM	9	0.5	1.8	0.00	0.1
	7	112	261	635	376
		8	73	6	89
REACTOME_REGULATION_OF_APOPTOSIS	5	0.6	1.7	0.02	0.1
	2	448	674	669	376
		1	17	4	96
CROONQUIST_NRAS_SIGNALING_DN	7	0.6	1.7	0.01	0.1
	0	864	747	652	378
		85	15	9	63
REACTOME_SIGNALING_BY_FGFR2_IIIA_TM	1	0.5	1.7	0.00	0.1
	9	906	366	619	378
		4	76	8	85
REACTOME_CYCLIN_A:CDK2_ASSOCIATED_EVENTS_AT_S_PHA	8	0.5	1.7	0.02	0.1
SE_ENTRY	5	775	811	531	379
		56	26	6	49

REACTOME_REGULATION_OF_PTEN_STABILITY_AND_ACTIVITY	68	0.539361	1.731848	0.036364	0.137979
REACTOME_NUCLEOBASE_BIOSYNTHESIS	15	0.690302	1.730002	0.018182	0.137989
BHAT_ESR1_TARGETS_VIA_AKT1_UP	277	0.451932	1.759273	0.004024	0.138006
REACTOME_RNA_POLYMERASE_II_PRE_TRANSCRIPTION_EVENTS	80	0.485191	1.73082	0.014	0.138152
REACTOME_MITOTIC_G2_G2_M_PHASES	198	0.464201	1.736998	0.010707	0.138564
REACTOME_PIWI_INTERACTING_RNA_PIRNA_BIOGENESIS	25	0.551692	1.726741	0.007921	0.138568
BURTON_ADIPOGENESIS_PEAK_AT_16HR	41	0.608618	1.76892	0.026369	0.138687
REACTOME_RNA_POLYMERASE_III_CHAIN_ELONGATION	18	0.641633	1.725697	0.012346	0.138697
REACTOME_METABOLISM_OF_POLYAMINES	58	0.618696	1.718563	0.031185	0.138729
LU_EZH2_TARGETS_UP	265	0.507021	1.775155	0.018036	0.138868
DACOSTA_UV_RESPONSE_VIA_ERCC3_UP	304	0.446879	1.719197	0.020492	0.138939
CHEMNITZ_RESPONSE_TO_PROSTAGLANDIN_E2_UP	139	0.513431	1.767539	0.018711	0.138969
REACTOME_TRANSCRIPTIONAL_REGULATION_BY_RUNX3	95	0.445382	1.731866	0.016736	0.139008
LE_NEURONAL_DIFFERENTIATION_DN	18	0.652263	1.73454	0.014	0.139268
ELVIDGE_HIF1A_AND_HIF2A_TARGETS_UP	40	0.528429	1.719829	0.028866	0.13929
REACTOME_TRANSLATION	2	0.5	1.7	0.04	0.1

	9	971	270	106	392
	1	85	15	8	93
SHEPARD_CRUSH_AND_BURN_MUTANT_UP	1	0.4	1.7	0.00	0.1
	9	348	279	829	393
	7	7	3	9	16
REACTOME_PTEN_REGULATION	1	0.4	1.7	0.01	0.1
	3	446	694	239	395
	6	86	9	7	08
REACTOME_HEDGEHOG_ON_STATE	8	0.4	1.7	0.01	0.1
	5	686	150	677	397
		85	57	1	63
WONG_EMBRYONIC_STEM_CELL_CORE	3	0.5	1.7	0.02	0.1
	2	705	756	714	397
	8	02	9		77
REACTOME_TRNA_PROCESSING_IN_THE_NUCLEUS	5	0.5	1.7	0.01	0.1
	7	720	321	417	397
		18	43		85
REACTOME_HIV_INFECTION	2	0.4	1.7	0.02	0.1
	2	660	200	132	400
	9	2	71	2	56
BASSO_B_LYMPHOCYTE_NETWORK	1	0.4	1.8	0.00	0.1
	4	869	261	642	400
	1	74	97	4	98
REACTOME_MITOTIC_G1_G1_S_PHASES	1	0.5	1.7	0.03	0.1
	4	379	208	601	401
	9	57	57	7	15
JAIN_NFKB_SIGNALING	7	0.4	1.7	<0.0	0.1
	2	678	766	1	402
		43	88		08
REACTOME_FGFR2_ALTERNATIVE_SPLICING	2	0.5	1.7	0.01	0.1
	7	716	163	214	403
		41	68	6	38
REACTOME_MITOCHONDRIAL_TRANSLATION	9	0.7	1.7	0.01	0.1
	4	001	136	960	403
		41	82	8	71
XU_CREBBP_TARGETS_UP	2	0.5	1.7	0.00	0.1
	1	423	125	809	406
		22	91	7	57
REACTOME_REGULATION_OF_RUNX3_EXPRESSION_AND_ACTI	5	0.6	1.7	0.02	0.1
VITY	5	241	697	282	407
		62	93	2	1
TONKS_TARGETS_OF_RUNX1_RUNX1T1_FUSION_MONOCYTE_U	2	0.4	1.7	0.00	0.1
P	0	015	150	847	407
	1	23	62	5	11
KEGG_PROTEASOME	4	0.6	1.7	0.03	0.1
	3	758	220	125	407

		94	72		69
DANG_MYC_TARGETS_UP	1	0.5	1.7	0.01	0.1
	2	788	774	709	408
	8	6	39	4	14
REACTOME_DEGRADATION_OF_BETA_CATENIN_BY_THE_DEST	8	0.5	1.7	0.03	0.1
RUCTION_COMPLEX	2	284	321	640	408
		56	64	3	59
REACTOME_VIRAL_MESSENGER_RNA_SYNTHESIS	4	0.5	1.7	0.02	0.1
	4	639	209	066	409
		04	7	1	77
REACTOME_HOST_INTERACTIONS_OF_HIV_FACTORS	1	0.5	1.7	0.02	0.1
	3	060	223	754	413
	0	27	76	2	93
REACTOME_TRANSCRIPTION_COUPLED_NUCLEOTIDE_EXCISIO	7	0.5	1.8	0.00	0.1
N_REPAIR_TC_NER	8	559	270	212	416
		7	38	3	62
RAMASWAMY_METASTASIS_UP	6	0.4	1.7	0.01	0.1
	4	734	040	821	417
		43	96	9	29
REACTOME_SCF_SKP2_MEDIATED_DEGRADATION_OF_P27_P21	6	0.6	1.7	0.01	0.1
	0	502	701	910	418
		86	7	8	4
NIKOLSKY_BREAST_CANCER_1Q21_AMPLICON	3	0.6	1.7	0.03	0.1
	7	331	045	984	421
		72	66	1	73
REACTOME_MRNA_DECAY_BY_3_TO_5_EXORIBONUCLEASE	1	0.6	1.6	0.01	0.1
	6	808	986	609	423
		6	56	7	87
POMEROY_MEDULLOBLASTOMA_PROGNOSIS_DN	4	0.6	1.6	0.02	0.1
	2	029	994	972	424
		63	28	4	
MORI_EMU_MYC_LYMPHOMA_BY_ONSET_TIME_UP	1	0.5	1.7	0.02	0.1
	0	022	101	464	424
	6	95	65	1	26
REACTOME_RRNA_PROCESSING	1	0.6	1.8	0.01	0.1
	9	332	283	836	425
	9	51	05	7	44
REACTOME_COMPLEX_I_BIOGENESIS	5	0.6	1.7	0.02	0.1
	5	770	000	674	425
		32	54	9	85
STARK_HYPPOCAMPUS_22Q11_DELETION_DN	1	0.7	1.7	0.01	0.1
	8	341	048	980	427
		38	62	2	3
REACTOME_EXTENSION_OF_TELOMERES	3	0.6	1.7	0.01	0.1
	0	581	019	419	432
		21	92	9	62

REACTOME_HIV_LIFE_CYCLE	1	0.4	1.7	0.03	0.1
	4	576	002	666	432
	8	5	44		77
MOOTHA_VOXPHOS	7	0.6	1.7	0.02	0.1
	2	728	085	658	432
		25	52	5	83
ACOSTA_PROLIFERATION_INDEPENDENT_MYC_TARGETS_UP	8	0.5	1.8	0.00	0.1
	3	237	295	203	434
		99	46	3	54
BURTON_ADIPOGENESIS_5	1	0.5	1.7	0.03	0.1
	0	215	009	658	434
	9	11	23	5	89
SOTIRIOU_BREAST_CANCER_GRADE_1_VS_3_UP	1	0.6	1.7	0.04	0.1
	5	604	049	421	435
	1	37	51	1	19
GARGALOVIC_RESPONSE_TO_OXIDIZED_PHOSPHOLIPIDS_TUR	5	0.5	1.8	0.00	0.1
QUOISE_DN	4	809	312	801	438
		58	7	6	61
GROSS_HYPOXIA_VIA_HIF1A_UP	7	0.4	1.7	0.01	0.1
	3	700	051	252	442
		87	43	6	28
KEGG_BASE_EXCISION_REPAIR	3	0.5	1.7	0.02	0.1
	3	750	055	626	447
		79	47	3	17
WEST_ADRENOCORTICAL_CARCINOMA_VS_ADENOMA_UP	1	0.5	1.6	0.02	0.1
	9	989	953	231	449
		86	11	2	35
REACTOME_ASSEMBLY_OF_THE_PRE_REPLICATIVE_COMPLEX	6	0.6	1.8	0.01	0.1
	7	683	322	260	455
		84	13	5	07
ZHAN_VARIABLE_EARLY_DIFFERENTIATION_GENES_DN	2	0.5	1.7	0.02	0.1
	9	532	056	386	455
		74	4	1	64
IRITANI_MAD1_TARGETS_DN	4	0.6	1.8	0.00	0.1
	1	498	337	8	461
		44	82		66
REACTOME_REGULATION_OF_MRNA_STABILITY_BY_PROTEIN	8	0.5	1.8	0.01	0.1
S_THAT_BIND_AU_RICH_ELEMENTS	6	799	346	295	479
		04	02	9	91
REACTOME_HIV_ELONGATION_ARREST_AND_RECOVERY	3	0.6	1.8	0.00	0.1
	2	206	363	603	485
		01	93	6	41
REACTOME_DEGRADATION_OF_GLI1_BY_THE_PROTEASOME	5	0.5	1.6	0.03	0.1
	8	808	903	651	488
		44	2	1	88
REACTOME_SIGNALING_BY_NOTCH4	8	0.4	1.6	0.02	0.1

	2	866	908	375	490
		81	92	8	54
BILD_MYC_ONCOGENIC_SIGNATURE	1	0.5	2.0	<0.0	0.1
	9	087	816	1	495
	5	96	9		34
REACTOME_FORMATION_OF_HIV_ELONGATION_COMPLEX_IN_	4	0.5	1.8	0.00	0.1
THE_ABSENCE_OF_HIV_TAT	4	888	367	828	511
		14	2	2	57
PID_MYC_ACTIV_PATHWAY	7	0.5	1.8	0.01	0.1
	9	321	445	455	528
		98	31	3	41
REACTOME_CDK_MEDIATED_PHOSPHORYLATION_AND_REMO	7	0.6	1.8	0.00	0.1
VAL_OF_CDC6	1	419	468	625	534
		71	7		14
NUTT_GBM_VS_AO_GLIOMA_DN	4	0.5	1.8	0.00	0.1
	4	487	372	400	535
		2	88	8	63
REACTOME_TRNA_PROCESSING	1	0.5	1.8	0.00	0.1
	0	832	410	410	545
	6	19	24	7	96
REACTOME_RNA_POLYMERASE_III_TRANSCRIPTION	4	0.5	1.8	0.00	0.1
	1	494	385	609	548
		65	78	8	06
REACTOME_FORMATION_OF_TUBULIN_FOLDING_INTERMEDIA	2	0.5	1.6	0.03	0.1
TES_BY_CCT_TRIC	6	721	820	080	548
		97	17	1	27
BENPORATH_MYC_TARGETS_WITH_EBOX	2	0.4	1.6	0.01	0.1
	2	009	832	057	551
	1	03	23	1	95
BARIS_THYROID_CANCER_UP	2	0.5	1.6	0.02	0.1
	4	571	820	096	556
		44	9	4	57
REACTOME_REGULATION_OF_RUNX2_EXPRESSION_AND_ACTI	7	0.5	1.6	0.03	0.1
VITY	2	239	834	347	558
		43	12	3	67
REACTOME_AUF1_HNRNP_D0_BINDS_AND_DESTABILIZES_MR	5	0.6	1.8	0.01	0.1
NA	4	821	469	263	570
		58		2	44
FRASOR_RESPONSE_TO_SERM_OR_FULVESTRANT_DN	4	0.7	1.9	<0.0	0.1
	8	333	946	1	570
		97	7		55
REACTOME_TRNA_MODIFICATION_IN_THE_NUCLEUS_AND_CY	4	0.6	1.8	0.00	0.1
TOSOL	2	263	489	409	578
		34	13	8	15
REACTOME_REGULATION_OF_RAS_BY_GAPS	6	0.5	1.6	0.03	0.1
	7	216	787	797	578

		72	2	5	15
REACTOME_RESPIRATORY_ELECTRON_TRANSPORT_ATP_SYNT	1	0.6	1.6	0.04	0.1
HESIS_BY_CHEMIOSMOTIC_COUPLING_AND_HEAT_PRODUCTI	0	251	766	123	584
ON_BY_UNCOUPLING_PROTEINS	5	59	58	7	67
AGUIRRE_PANCREATIC_CANCER_COPY_NUMBER_UP	2	0.4	1.6	0.01	0.1
	8	266	772	574	586
	8	8	67	8	49
CHICAS_RB1_TARGETS_LOW_SERUM	8	0.4	1.6	0.02	0.1
	8	879	755	306	588
		87	07	1	93
REACTOME_THE_ROLE_OF_GTSE1_IN_G2_M_PROGRESSION_AF	7	0.6	1.8	0.01	0.1
TER_G2_CHECKPOINT	7	162	530	271	590
		9	69	2	33
REACTOME_SWITCHING_OF_ORIGINS_TO_A_POST_REPLICATIV	8	0.6	1.8	0.00	0.1
E_STATE	9	386	501	626	598
		69	49	3	35
WHITEFORD_PEDIATRIC_CANCER_MARKERS	1	0.5	1.6	0.04	0.1
	1	829	733	573	605
	4	83	23	8	48
REACTOME_CELLULAR_RESPONSE_TO_HYPOXIA	7	0.5	1.6	0.04	0.1
	4	291	685	228	606
		29	85	3	69
CHAUHAN_RESPONSE_TO_METHOXYESTRADIOL_UP	4	0.6	2.0	0.00	0.1
	9	252	041	205	607
		95	13	3	4
REACTOME_ABORTIVE_ELONGATION_OF_HIV_1_TRANSCRIPT_	2	0.7	1.8	<0.0	0.1
IN_THE_ABSENCE_OF_TAT	3	033	685	1	610
		7	85		09
BAKKER_FOXO3_TARGETS_DN	1	0.3	1.6	0.00	0.1
	6	671	705	409	611
	4	42	23	8	88
REACTOME_TRISTETRAPROLIN_TTP_ZFP36_BINDS_AND_DESTA	1	0.6	1.6	0.03	0.1
BILIZES_MRNA	7	029	719	206	613
		95	49	4	1
REACTOME_CROSS_PRESENTATION_OF_SOLUBLE_EXOGENOU	4	0.6	1.6	0.04	0.1
S_ANTIGENS_ENDOSOMES	8	100	694	722	614
		7	34	8	89
REACTOME_ABC_TRANSPORTER_DISORDERS	7	0.5	1.6	0.03	0.1
	6	057	686	885	615
		53	23	5	02
KEGG_SPLICEOSOME	1	0.5	1.8	0.01	0.1
	2	799	538	446	620
	6	36	1	3	54
AMIT_SERUM_RESPONSE_480_MCF10A	3	0.5	1.6	0.01	0.1
	7	132	658	980	621
		72	11	2	82

BHAT_ESR1_TARGETS_NOT_VIA_AKT1_UP	2	0.3	1.6	0.00	0.1
	0	983	665	193	622
	7	81	47	1	09
ODONNELL_METASTASIS_DN	2	0.6	1.8	<0.0	0.1
	4	191	706	1	627
		63	34		48
YAO_TEMPORAL_RESPONSE_TO_PROGESTERONE_CLUSTER_14	1	0.4	1.6	0.02	0.1
	3	865	629	412	629
	8	14	42	3	09
PEART_HDAC_PROLIFERATION_CLUSTER_DN	7	0.5	1.8	0.00	0.1
	4	234	737	423	630
		28	1	7	78
REACTOME_RNA_POLYMERASE_II_TRANSCRIPTION_TERMINA	6	0.5	1.6	0.03	0.1
TION	5	422	630	272	636
		68	78		7
REACTOME_PROCESSING_OF_CAPPED_INTRON_CONTAINING_P	2	0.5	1.8	0.00	0.1
RE_MRNA	3	569	761	816	640
	7	2	7	3	74
REACTOME_KSRP_KHSRP_BINDS_AND_DESTABILIZES_MRNA	1	0.6	1.8	<0.0	0.1
	7	854	578	1	641
		76	47		02
REACTOME_RESPIRATORY_ELECTRON_TRANSPORT	9	0.6	1.6	0.04	0.1
	9	363	632	347	644
		56	09	8	09
MOREIRA_RESPONSE_TO_TSA_UP	2	0.6	1.8	0.00	0.1
	7	430	820	204	651
		63	99	9	35
PID_MYC_PATHWAY	2	0.5	1.8	0.00	0.1
	5	899	628	421	655
		72	39	1	75
SANSOM_APC_MYC_TARGETS	2	0.4	1.8	0.00	0.1
	3	650	541	407	660
	2	42	63	3	02
REACTOME_MICRORNA_MIRNA_BIOGENESIS	2	0.5	1.6	0.01	0.1
	4	506	583	596	660
		08	28	8	79
REACTOME_INFECTIOUS_DISEASE	3	0.4	1.6	0.03	0.1
	7	530	590	871	660
	5	9	08		96
REACTOME_DNA_STRAND_ELONGATION	3	0.6	1.6	0.03	0.1
	2	793	565	278	665
		5	32	7	12
SHIPP_DLBCL_VS_FOLLICULAR_LYMPHOMA_UP	4	0.6	1.6	0.03	0.1
	4	183	567	991	671
		8	49	1	06
BURTON_ADIPOGENESIS_PEAK_AT_24HR	4	0.5	1.6	0.04	0.1

	4	914	530	480	673
		64	11	7	16
NIKOLSKY_BREAST_CANCER_7P22_AMPLICON	3	0.6	1.6	0.04	0.1
	6	642	541	356	676
		18	47	4	
KEGG_RNA_POLYMERASE	2	0.6	1.9	<0.0	0.1
	9	841	803	1	676
		37	55		48
SPIELMAN_LYMPHOBLAST_EUROPEAN_VS_ASIAN_UP	4	0.4	1.8	0.00	0.1
	6	837	771	414	681
	8	79	96	1	21
SMID_BREAST_CANCER_LUMINAL_A_DN	1	0.7	1.8	0.00	0.1
	8	862	582	419	682
		12	64	3	84
REACTOME_APC_C:CDH1_MEDIATED_DEGRADATION_OF_CDC2	7	0.6	1.8	0.00	0.1
0_AND_OTHER_APC_C:CDH1_TARGETED_PROTEINS_IN_LATE_	1	693	835	418	683
MITOSIS_EARLY_G1		44	31	4	71
REACTOME_TRANSCRIPTIONAL_REGULATION_BY_RUNX2	1	0.4	1.6	0.03	0.1
	1	114	503	340	689
	7	5	51	3	41
BURTON_ADIPOGENESIS_4	4	0.5	1.6	0.02	0.1
	3	029	509	731	690
		9	56	1	04
STARK_PREFRONTAL_CORTEX_22Q11_DELETION_DN	4	0.4	1.6	0.03	0.1
	8	649	450	983	691
	4	02	29	2	75
ELVIDGE_HYPOXIA_DN	1	0.4	1.6	0.04	0.1
	4	431	456	184	693
	5	33	89	1	37
DAIRKEE_CANCER_PRONE_RESPONSE_BPA_E2	1	0.4	1.6	0.02	0.1
	1	162	469	310	694
	4	38	58	9	94
REACTOME_FGFR2_MUTANT_RECEPTOR_ACTIVATION	3	0.4	1.6	0.01	0.1
	1	969	489	006	696
		92	89		11
KIM_GASTRIC_CANCER_CHEMOSENSITIVITY	8	0.4	1.6	0.01	0.1
	8	409	424	388	697
		48	39	9	23
MULLIGAN_NTF3_SIGNALING_VIA_INSR_AND_IGF1R_UP	2	0.5	1.6	0.03	0.1
	2	619	459	829	697
		36	68	8	57
SMID_BREAST_CANCER_RELAPSE_IN_BRAIN_UP	3	0.4	1.6	0.01	0.1
	9	777	428	195	701
		74	72	2	08
REACTOME_FANCONI_ANEMIA_PATHWAY	3	0.6	1.8	0.00	0.1
	9	112	898	205	703

		51	61	3	32
NAGY_TFTC_COMPONENTS_HUMAN	1	0.6	1.8	0.00	0.1
	9	549	857	407	705
		45	63	3	02
COLDREN_GEFITINIB_RESISTANCE_UP	8	0.4	1.6	0.01	0.1
	2	217	394	616	709
		4	15	2	87
REACTOME_MRNA_SPLICING	1	0.5	1.9	0.00	0.1
	8	824	231	811	732
	6	55	59	4	76
BONOME_OVARIAN_CANCER_POOR_SURVIVAL_DN	2	0.6	1.6	0.03	0.1
	0	199	342	092	740
		22	71	8	76
REACTOME_MTORC1_MEDIATED_SIGNALLING	2	0.5	1.6	0.03	0.1
	2	276	334	658	743
		3	59	5	47
REACTOME_DNA_DAMAGE_RECOGNITION_IN_GG_NER	3	0.6	2.0	<0.0	0.1
	8	398	108	1	744
		77	39		96
CUI_TCF21_TARGETS_2_UP	4	0.3	1.6	0.00	0.1
	0	759	310	613	749
	5	56	93	5	33
SONG_TARGETS_OF_IE86_CMV_PROTEIN	5	0.6	1.6	0.04	0.1
	5	099	315	842	750
		36	45	1	9
SCHUHMACHER_MYC_TARGETS_UP	7	0.6	1.9	<0.0	0.1
	9	721	688	1	756
		55	41		88
CHOW_RASSF1_TARGETS_DN	3	0.5	1.6	0.02	0.1
	0	216	294	966	760
		03	8	1	57
COLLER_MYC_TARGETS_UP	2	0.7	1.8	0.00	0.1
	4	244	901	211	771
		59	07		36
WELCSH_BRCA1_TARGETS_DN	1	0.6	2.0	<0.0	0.1
	3	202	260	1	772
	9	21	83		94
REACTOME_RNA_POLYMERASE_I_TRANSCRIPTION_INITIATIO	4	0.5	1.9	0.00	0.1
N	7	929	159	801	780
		48	74	6	77
REACTOME_FORMATION_OF_THE_EARLY_ELONGATION_COMP	3	0.6	1.9	0.00	0.1
LEX	3	543	108	209	781
		06	2	6	63
REACTOME_RNA_POLYMERASE_II_TRANSCRIPTION_PRE_INITI	4	0.5	1.6	0.02	0.1
ATION_AND_PROMOTER_OPENING	7	147	259	459	788
		75	06		79

PID_RB_1PATHWAY	6<br>4	0.4<br>148<br>97	1.6<br>251<br>28	0.02<br>012<br>1	0.1<br>790<br>19
REACTOME_MRNA_SPLICING_MINOR_PATHWAY	5<br>2	0.6<br>973<br>51	2.0<br>434<br>35	<0.0<br>1	0.1<br>797<br>15
REACTOME_FORMATION_OF_TC_NER_PRE_INCISION_COMPLE<br>X	5<br>3	0.6<br>087<br>14	1.9<br>250<br>28	0.00<br>211<br>9	0.1<br>804<br>06
LI_DCP2_BOUND_MRNA	8<br>6	0.6<br>625<br>8	1.8<br>921<br>45	0.00<br>826<br>4	0.1<br>804<br>13
REACTOME_RRNA_MODIFICATION_IN_THE_NUCLEUS_AND_CY<br>TOSOL	5<br>9	0.6<br>773<br>77	1.9<br>041<br>73	0.00<br>409<br>8	0.1<br>824<br>26
REACTOME_RNA_POLYMERASE_I_TRANSCRIPTION_TERMINAT<br>ION	3<br>1	0.6<br>447<br>44	1.9<br>291<br>41	0.00<br>195<br>3	0.1<br>839<br>08
REACTOME_NUCLEOTIDE_EXCISION_REPAIR	1<br>1<br>0	0.5<br>392<br>23	1.8<br>936<br>2	0.00<br>208<br>8	0.1<br>860<br>06
BIOCARTA_MCM_PATHWAY	1<br>8	0.6<br>962<br>5	1.6<br>182<br>99	0.03<br>178	0.1<br>868<br>88
DACOSTA_UV_RESPONSE_VIA_ERCC3_TTD_UP	5<br>9	0.5<br>099<br>61	1.6<br>174<br>59	0.03<br>673<br>5	0.1<br>870<br>47
ELVIDGE_HYPOXIA_BY_DMOG_DN	5<br>9	0.4<br>569<br>41	1.6<br>160<br>8	0.03<br>048<br>8	0.1<br>879<br>35
MANALO_HYPOXIA_DN	2<br>8<br>7	0.6<br>445<br>97	1.9<br>543<br>55	0.00<br>205<br>3	0.1<br>886<br>99
DANG_REGULATED_BY_MYC_UP	6<br>7	0.5<br>679<br>88	1.8<br>966<br>53	0.00<br>213<br>2	0.1<br>899<br>9
LIN_APC_TARGETS	7<br>0	0.5<br>227<br>9	1.6<br>127<br>83	0.04<br>536<br>1	0.1<br>904<br>24
ZHANG_RESPONSE_TO_CANTHARIDIN_DN	6<br>3	0.6<br>793<br>5	1.9<br>368<br>68	<0.0<br>1	0.1<br>943<br>29
SCHLOSSER_MYC_AND_SERUM_RESPONSE_SYNERGY	3<br>1	0.7<br>387<br>82	1.9<br>293<br>83	0.00<br>407<br>3	0.1<br>975<br>34
MELLMAN_TUT1_TARGETS_UP	2	0.5	1.6	0.02	0.2

	0	441	037	898	003
		35	32	6	26
ZAMORA_NOS2_TARGETS_UP	5	0.5	1.5	0.03	0.2
	0	036	998	640	029
		9	38	3	69
MUELLER_PLURINET	2	0.5	1.9	0.00	0.2
	9	704	404	417	029
	6	07	81	5	95
REACTOME_APC_C:CDC20_MEDIATED_DEGRADATION_OF_CYC	2	0.6	1.5	0.03	0.2
LIN_B	3	057	984	838	038
		99	92		35
KYNG_RESPONSE_TO_H2O2_VIA_ERCC6_DN	4	0.4	1.5	0.02	0.2
	4	600	969	390	042
		96	24	4	56
WANG_RESPONSE_TO_BEXAROTENE_UP	3	0.4	1.5	0.03	0.2
	2	597	899	448	116
		54	32	3	16
REACTOME_BETA_CATENIN_INDEPENDENT_WNT_SIGNALING	1	0.3	1.5	0.02	0.2
	4	717	848	526	123
	1	7	02	3	24
REACTOME_DUAL_INCISION_IN_GG_NER	4	0.5	1.5	0.04	0.2
	1	191	850	651	137
		82	4	2	09
RIZ_ERYTHROID_DIFFERENTIATION_CCNE1	4	0.4	1.5	0.03	0.2
	3	520	748	621	168
		22	16	7	36
REACTOME_TRANSCRIPTIONAL_REGULATION_BY_TP53	3	0.3	1.5	0.02	0.2
	5	668	651	822	206
	5	44	52	6	92
JAZAERI_BREAST_CANCER_BRCA1_VS_BRCA2_UP	4	0.4	1.5	0.03	0.2
	8	375	673	420	210
		5	59	5	57
KEGG_PURINE_METABOLISM	1	0.3	1.5	0.02	0.2
	5	660	606	272	213
	1	41	12	7	06
REACTOME_DEADENYLATION_DEPENDENT_MRNA_DECAY	5	0.4	1.5	0.04	0.2
	3	750	677	480	213
		84	83	7	48
MOOTHA_PGC	4	0.3	1.5	0.04	0.2
	1	997	622	564	214
	0	69	21	3	83
KYNG_RESPONSE_TO_H2O2_VIA_ERCC6	1	0.5	1.5	0.03	0.2
	7	692	680	232	218
		28	17	8	28
REACTOME_METABOLISM_OF_NUCLEOTIDES	9	0.4	1.5	0.03	0.2
	8	236	685	278	219

		64	75	7	14
KEGG_GLUTATHIONE_METABOLISM	4	0.4	1.5	0.04	0.2
	4	904	460	958	297
		81	34	7	44
DAIRKEE_CANCER_PRONE_RESPONSE_BPA	4	0.4	1.5	0.03	0.2
	9	441	469	711	299
		14	86	3	99
REACTOME_NEDDYLATION	2	0.3	1.5	0.04	0.2
	2	668	353	801	372
	7	37	74	7	34
SANSOM_APC_TARGETS_REQUIRE_MYC	2	0.5	2.0	<0.0	0.2
	1	760	955	1	387
	2	39	72		91

Table S6. GSEA analysis between low- and high-STXBP5-AS1 phenotype using the c5 reference gene set.

NAME	SIZE	ES	NES	NOM p-val	FDR q-val
GO_SNRNA_BINDING	38	0.688476	2.191844	<0.01	0.023358
GO_PYRIMIDINE_RIBONUCLEOTIDE_BIOSYNTHETIC_PROCESS	21	0.695766	2.009705	<0.01	0.145312
GO_MATURATION_OF_SSU_RRNA	44	0.680115	1.945564	<0.01	0.149607
GO_SM_LIKE_PROTEIN_FAMILY_COMPLEX	73	0.638351	1.951033	0.001912	0.151183
GO_PROTEIN_TRANSMEMBRANE_IMPORT_INTO_INTRACELLULAR_ORGANELLE	34	0.668704	1.924995	<0.01	0.155307
GO_MATURATION_OF_SSU_RRNA_FROM_TRICISTRONIC_RRNA_TRANSCRIPT_SSU_RRNA_5_8S_RRNA_LSU_RRNA_	34	0.717721	1.971195	<0.01	0.155886
GO_PRERIBOSOME	73	0.675908	1.962094	<0.01	0.157117
GO_SPLICEOSOMAL_TRI_SNRNP_COMPLEX	31	0.738948	1.954518	<0.01	0.157632
GO_90S_PRERIBOSOME	30	0.696949	1.934656	0.001965	0.158239
GO_RNA_POLYMERASE_ACTIVITY	43	0.66966	2.059933	<0.01	0.161371
GO_TRANSCRIPTION_INITIATION_FROM_RNA_POLYMERASE_I_PROMOTER	35	0.643267	1.926191	<0.01	0.163581
GO_RRNA_METABOLIC_PROCESS	200	0.599718	1.864567	0.005837	0.164423
GO_NCRNA_METABOLIC_PROCESS	431	0.545491	1.867652	0.005814	0.165137
GO_NUCLEOLAR_PART	180	0.46551	1.87030	<0.01	0.165573

		5	5		
GO_PRECATALYTIC_SPLICEOSOME	44	0.662708	1.861034	0.007634	0.165622
GO_TRNA_WOBBLE_URIDINE_MODIFICATION	15	0.722483	1.835944	0.003831	0.169214
GO_PROTEIN_IMPORT_INTO_MITOCHONDRIAL_MATRIX	19	0.770095	1.870926	<0.01	0.170819
GO_PYRIMIDINE_NUCLEOSIDE_TRIPHOSPHATE_BIOSYNTHETIC_PROCESS	18	0.741164	2.013387	<0.01	0.171966
GO_GLYCOSYL_COMPOUND_BIOSYNTHETIC_PROCESS	41	0.59893	1.973117	<0.01	0.173565
GO_NUCLEAR_EXOSOME_RNASE_COMPLEX_	16	0.769242	1.835994	0.005952	0.173594
GO_DNA_HELICASE_COMPLEX	15	0.696986	1.853334	<0.01	0.175711
GO_NUCLEOTIDE_EXCISION_REPAIR_DNA_DAMAGE_RECOGNITION	23	0.67275	1.871761	0.003861	0.175971
GO_HEMATOPOIETIC_STEM_CELL_DIFFERENTIATION	77	0.520097	1.837081	0.007692	0.1764
GO_MATURATION_OF_5_8S_RRNA	26	0.708848	1.839417	0.001946	0.176827
GO_EXOSOME_RNASE_COMPLEX_	25	0.705884	1.983289	<0.01	0.177575
GO_RIBOSOME_BIOGENESIS	272	0.59937	1.873635	0.007782	0.179164
GO_POSITIVE_REGULATION_OF_VIRAL_TRANSCRIPTION	41	0.542347	1.840167	0.003906	0.180649
GO_TRANSCRIPTION_BY_RNA_POLYMERASE_I	62	0.558457	1.845258	0.001969	0.18165
GO_RIBOSOMAL_LARGE_SUBUNIT_BIOGENESIS	68	0.681465	1.841896	0.007722	0.182396

GO_SMALL_SUBUNIT_PROCESSOME	37	0.66673	1.846027	0.005837	0.185772
GO_SMALL_NUCLEOLAR_RIBONUCLEOPROTEIN_COMPLEX	22	0.777585	1.824241	0.00198	0.185903
GO_TRANSCRIPTION_BY_RNA_POLYMERASE_III	54	0.522303	1.873907	0.004	0.18646
GO_RIBOSOMAL_SMALL_SUBUNIT_BIOGENESIS	62	0.665264	1.894611	0.001898	0.187113
GO_RIBONUCLEOPROTEIN_COMPLEX_BIOGENESIS	429	0.532261	1.812995	0.019048	0.189473
GO_RRNA_TRANSCRIPTION	30	0.629586	1.902701	0.004024	0.191544
GO_RNA_POLYMERASE_III_ACTIVITY	15	0.716239	1.818841	<0.01	0.191709
GO_SNORNA_BINDING	24	0.734579	1.897285	<0.01	0.192475
GO_ANAPHASE_PROMOTING_COMPLEX_DEPENDENT_CATABOLIC_PROCESS	80	0.644362	1.813479	0.017442	0.192866
GO_SPLICEOSOMAL_SNRNP_ASSEMBLY	38	0.662057	1.874618	0.001957	0.193256
GO_NCRNA_PROCESSING	358	0.559891	1.881987	0.005814	0.195109
GO_TRANSCRIPTION_COUPLED_NUCLEOTIDE_EXCISION_REPAIR	73	0.560487	1.814113	<0.01	0.196274
GO_TERMINATION_OF_RNA_POLYMERASE_I_TRANSCRIPTION	31	0.635174	1.806714	0.006	0.197055
GO_RNA_POLYMERASE_COMPLEX	105	0.49385	1.779772	0.007782	0.197916
GO_MATURATION_OF_5_8S_RRNA_FROM_TRICISTRONIC_RRNA_TRANSCRIPT_SSU_RRNA_5_8S_RRNA_LSU_RRNA_	21	0.710824	1.77753	0.00578	0.19856
GO_PURINE_NUCLEOSIDE_BIOSYNTHETIC_PROCESS	22	0.65	1.87	<0.01	0.198

		7612	6146		638
GO_RNA_MODIFICATION	154	0.503632	1.782913	0.008016	0.198905
GO_ORGANELLAR_RIBOSOME	87	0.709871	1.775281	0.005682	0.199611
GO_RNA_POLYMERASE_II_CORE_COMPLEX	15	0.689079	1.796318	0.011278	0.200179
GO_U12_TYPE_SPLICEOSOMAL_COMPLEX	26	0.674164	1.784103	0.009728	0.200273
GO_NUCLEOBASE_BIOSYNTHETIC_PROCESS	19	0.721503	1.800285	0.01	0.200524
GO_CAJAL_BODY	56	0.550355	1.780206	0.014344	0.200572
GO_U2_TYPE_SPLICEOSOMAL_COMPLEX	81	0.572537	1.789228	0.021113	0.202279
GO_MITOCHONDRIAL_RESPIRATORY_CHAIN_COMPLEX_ASSEMBLY	94	0.681896	1.80138	0.015656	0.202737
GO_ANAPHASE_PROMOTING_COMPLEX	21	0.664649	1.797169	0.008016	0.202871
GO_TRNA_PROCESSING	125	0.575968	1.784506	0.008065	0.203279
GO_O_METHYLTRANSFERASE_ACTIVITY	24	0.623112	1.882734	0.00396	0.203622
GO_PRERIBOSOME_LARGE_SUBUNIT_PRECURSOR	22	0.804208	2.020507	<0.01	0.204601
GO_TRNA_METABOLIC_PROCESS	172	0.565346	1.789844	0.023529	0.205218
GO_PYRIMIDINE_NUCLEOSIDE_MONOPHOSPHATE_BIOSYNTHETIC_PROCESS	16	0.665519	1.784761	0.001927	0.206568
GO_CHAPERONE_COMPLEX	20	0.71538	1.79113	0.009597	0.206827

		6	8		
GO_MITOCHONDRIAL_TRANSLATIONAL_TERMINATION	89	0.711573	1.765056	0.011278	0.215302
GO_PYRIMIDINE_NUCLEOTIDE_BIOSYNTHETIC_PROCESS	45	0.511612	1.759908	0.007339	0.221918
GO_MATURATION_OF_LSU_RRNA	20	0.724823	1.756371	0.009452	0.221995
GO_BASE_EXCISION_REPAIR	41	0.547953	1.703095	0.030242	0.223309
GO_TRANSLATIONAL_TERMINATION	104	0.678161	1.7572	0.022642	0.223756
GO_ORGANELLE_INNER_MEMBRANE	483	0.520542	1.75349	0.015414	0.224028
GO_CATALYTIC_ACTIVITY_ACTING_ON_RNA	335	0.457972	1.703808	0.021611	0.224134
GO_RIBONUCLEOSIDE_TRIPHOSPHATE_BIOSYNTHETIC_PROCESS	43	0.524221	1.704722	0.012939	0.224864
GO_NADH_DEHYDROGENASE_COMPLEX_ASSEMBLY	62	0.676698	1.70566	0.021154	0.225371
GO_NUCLEOTIDE_EXCISION_REPAIR	107	0.496709	1.749306	0.015595	0.225731
GO_NUCLEOSIDE_MONOPHOSPHATE_BIOSYNTHETIC_PROCESS	43	0.539568	1.706516	0.019084	0.226
GO_ORGANELLAR_LARGE_RIBOSOMAL_SUBUNIT	57	0.718552	1.711161	0.013619	0.227215
GO_MITOCHONDRIAL_GENE_EXPRESSION	160	0.646436	1.749951	0.017682	0.227704
GO_TRNA_WOBBLE_BASE_MODIFICATION	19	0.643057	1.709524	0.011788	0.227823
GO_REGULATION_OF_ESTABLISHMENT_OF_PLANAR_POLARITY	108	0.434299	1.706535	0.012072	0.228307

GO_REGULATION_OF_HEMATOPOIETIC_PROGENITOR_CELL_DIFFERENTIATION	81	0.471775	1.713017	0.026415	0.228416
GO_RNA_SPLICING_VIA_TRANSESTERIFICATION_REACTIONS	320	0.480828	1.707621	0.037255	0.228761
GO_RIBONUCLEOSIDE_MONOPHOSPHATE_BIOSYNTHETIC_PROCESS	32	0.562703	1.715225	0.017274	0.229011
GO_LARGE_RIBOSOMAL_SUBUNIT	116	0.672051	1.716307	0.028791	0.229366
GO_RIBOSOME	223	0.609492	1.711302	0.039848	0.229418
GO_NUCLEOTIDE_EXCISION_REPAIR_DNA_DUPLEX_UNWINDING	22	0.62077	1.745719	0.013462	0.229837
GO_VIRAL_GENE_EXPRESSION	190	0.539839	1.713258	0.03301	0.230478
GO_TELOMERASE_HOLOENZYME_COMPLEX	20	0.618981	1.724933	0.019685	0.231387
GO_TRANSCRIPTION_ELONGATION_FROM_RNA_POLYMERASE_I_PROMOTER	30	0.614019	1.716501	0.017822	0.231624
GO_DNA_TEMPLATED_TRANSCRIPTION_ELONGATION	107	0.487852	1.741006	0.021401	0.232431
GO_TRNA_MODIFICATION	83	0.565756	1.742518	0.025743	0.23272
GO_NEGATIVE_REGULATION_OF_CELL_CYCLE_G2_M_PHASE_TRANSITION	98	0.520075	1.73753	0.021236	0.233286
GO_RIBOSOMAL_LARGE_SUBUNIT_ASSEMBLY	29	0.683097	1.716871	0.011364	0.233487
GO_REGULATION_OF_TRANSCRIPTION_BY_RNA_POLYMERASE_I	32	0.545769	1.692471	0.027613	0.233563
GO_CATALYTIC_ACTIVITY_ACTING_ON_A_TRNA	119	0.546635	1.725198	0.027613	0.233682
GO_MITOCHONDRIAL_TRANSLATION	13	0.65	1.73	0.023	0.234

	5	9846	8686	121	187
GO_DNA_REPLICATION_INITIATION	36	0.677793	1.733808	0.035928	0.234501
GO_PROTEASOMAL_UBIQUITIN_INDEPENDENT_PROTEIN_CATABOLIC_PROCESS	22	0.703586	1.694244	0.025391	0.234674
GO_TRANSLATIONAL_ELONGATION	131	0.593585	1.690684	0.037879	0.234692
GO_NUCLEOTIDYLTRANSFERASE_ACTIVITY	115	0.469012	1.717352	0.01004	0.235294
GO_PEPTIDASE_COMPLEX	85	0.533788	1.689085	0.036122	0.235378
GO_REGULATION_OF_CELLULAR_AMINE_METABOLIC_PROCESS	77	0.522699	1.725764	0.033138	0.235602
GO_CLEAVAGE_INVOLVED_IN_RRNA_PROCESSING	21	0.685758	1.692564	0.019048	0.235669
GO_INNER_MITOCHONDRIAL_MEMBRANE_PROTEIN_COMPLEX	110	0.620318	1.677508	0.02947	0.235727
GO_NUCLEAR_TRANSCRIBED_MRNA_CATABOLIC_PROCESS_EXONUCLEOLYTIC	35	0.587861	1.670385	0.035928	0.235757
GO_RNA_SPLICING	404	0.46884	1.71968	0.029644	0.235957
GO_REGULATION_OF_STEM_CELL_DIFFERENTIATION	109	0.407877	1.671301	0.016981	0.23598
GO_RRNA_MODIFICATION	33	0.585858	1.667149	0.031936	0.236083
GO_NCRNA_3_END_PROCESSING	36	0.56547	1.665146	0.031311	0.236299
GO_RNA_5_END_PROCESSING	21	0.66341	1.668978	0.031434	0.236501
GO_REGULATION_OF_CELLULAR_AMINO_ACID_METABOLIC_PROCESS	59	0.574888	1.665966	0.040936	0.236582

GO_TRNA_SPECIFIC_RIBONUCLEASE_ACTIVITY	16	0.691278	1.678109	0.021696	0.236586
GO_RNA_POLYMERASE_III_COMPLEX	17	0.646652	1.726818	0.009766	0.236676
GO_REGULATION_OF_TRANSCRIPTION_ELONGATION_FROM_RNA_POLYMERASE_II_PROMOTER	29	0.542576	1.694387	0.010309	0.23672
GO_U1_SNRNP	18	0.682185	1.667647	0.019802	0.237092
GO_REGULATION_OF_VIRAL_TRANSCRIPTION	64	0.45447	1.671645	0.010081	0.237289
GO_SPLICEOSOMAL_COMPLEX	170	0.528206	1.733996	0.037698	0.237383
GO_NCRNA_TRANSCRIPTION	102	0.461543	1.686605	0.029183	0.237562
GO_DNA_TEMPLATED_TRANSCRIPTION_TERMINATION	74	0.533837	1.680772	0.031373	0.237708
GO_MITOCHONDRIAL_MEMBRANE_ORGANIZATION	107	0.459694	1.71741	0.009416	0.237982
GO_REGULATION_OF_TRANSCRIPTION_BY_RNA_POLYMERASE_III	23	0.526267	1.678462	0.012048	0.237999
GO_ENDOPEPTIDASE_COMPLEX	63	0.575768	1.6633	0.045977	0.238143
GO_RNA_3_END_PROCESSING	134	0.488684	1.719962	0.02924	0.238235
GO_INO80_TYPE_COMPLEX	24	0.555111	1.671837	0.020202	0.238832
GO_RIBONUCLEOPROTEIN_COMPLEX_SUBUNIT_ORGANIZATION	239	0.468496	1.681181	0.034286	0.239006
GO_INTERCELLULAR_BRIDGE	56	0.444782	1.727175	0.001938	0.239018
GO_SNRNA_PROCESSING	28	0.56	1.67	0.009	0.239

		6594	4698	69	211
GO_NEGATIVE_REGULATION_OF_MRNA_SPLICING_VIA_SPLICEOSOME	20	0.61039	1.682302	0.038536	0.239358
GO_MITOCHONDRION_ORGANIZATION	485	0.412614	1.683198	0.013283	0.239828
GO_PSEUDOURIDINE_SYNTHESIS	17	0.712075	1.684348	0.007828	0.239841
GO_MITOCHONDRIAL_PROTEIN_COMPLEX	237	0.621109	1.729661	0.023301	0.23989
GO_LIGASE_ACTIVITY_FORMING_CARBON_NITROGEN_BONDS	42	0.496437	1.660334	0.023576	0.240094
GO_RIBOSOME_ASSEMBLY	59	0.634977	1.7281	0.021154	0.240215
GO_ORGANELLE_ENVELOPE_LUMEN	85	0.502302	1.672114	0.026923	0.240342
GO_RNA_CATABOLIC_PROCESS	376	0.439263	1.672715	0.028958	0.241227
GO_PROTEIN_INSERTION_INTO_MEMBRANE	59	0.466837	1.657357	0.011538	0.242197
GO_U2_SNRNP	19	0.658314	1.656071	0.028355	0.242713
GO_REGULATION_OF_MITOCHONDRIAL_TRANSLATION	25	0.651435	1.65491	0.044146	0.242896
GO_NUCLEOBASE_CONTAINING_SMALL_MOLECULE_BIOSYNTHETIC_PROCESS	98	0.404546	1.652604	0.003824	0.243529
GO_REGULATION_OF_TRANSCRIPTION_FROM_RNA_POLYMERASE_II_PROMOTER_IN_RESPONSE_TO_HYPOXIA	75	0.504914	1.657555	0.044316	0.243617
GO_OUTER_MITOCHONDRIAL_MEMBRANE_PROTEIN_COMPLEX	16	0.625178	1.651419	0.031955	0.243984

Table S7. DEG between high- and low-STXBP5-AS1 expression groups in COAD.

ID	logFC	PValue	FDR	ID	logFC	PValue	FDR
HIST1H1E	-5.609792	1.73E-73	3.03E-69	KRT6A	-1.812132	4.62E-10	1.71E-08
HIST1H1D	-5.438048	2.48E-68	2.18E-64	CBLN4	1.6715587	4.83E-10	1.78E-08
HIST1H1B	-5.887735	3.44E-67	2.01E-63	LCE3D	-2.557135	4.84E-10	1.78E-08
HIST1H4C	-6.048038	5.54E-65	2.43E-61	PAK5	2.4169058	5.18E-10	1.88E-08
HIST1H3B	-5.679372	3.35E-63	1.17E-59	FABP2	-1.083166	5.19E-10	1.88E-08
HIST1H4D	-5.477742	1.10E-61	3.22E-58	IL17C	1.2734573	5.39E-10	1.94E-08
HIST1H4E	-4.485252	3.09E-60	7.75E-57	CHRNB2	1.383772	5.73E-10	2.06E-08
HIST1H2AI	-4.14767	4.05E-58	8.88E-55	CD70	1.0854033	5.94E-10	2.12E-08
HIST1H2BO	-4.6248	6.39E-56	1.24E-52	KRT81	1.1764014	6.00E-10	2.14E-08
HIST1H2AJ	-5.03882	1.35E-55	2.37E-52	HIST3H2BB	-1.01346	6.39E-10	2.25E-08
HIST1H2AD	-3.760225	9.04E-55	1.44E-51	PCDHGA8	-1.190256	7.36E-10	2.55E-08
HIST1H2AM	-3.81065	2.24E-53	3.27E-50	GIF	-1.332039	7.92E-10	2.72E-08
HIST1H3F	-4.808401	1.34E-50	1.81E-47	FGF8	1.2527451	8.18E-10	2.79E-08
PAEP	5.4060472	1.74E-50	2.18E-47	AC009163.2	-1.229019	8.26E-10	2.82E-08
HIST1H2BL	-3.756465	3.07E-50	3.59E-47	SLC4A10	-1.311499	9.17E-10	3.09E-08
HIST1H2BI	-5.795868	7.52E-49	8.23E-46	RETNLB	-1.358993	1.04E-09	3.45E-08
HIST1H2AH	-4.54805	4.92E-47	5.07E-44	JPH3	1.0115452	1.04E-09	3.45E-08
HIST1H3A	-4.222472	1.87E-46	1.82E-43	ADAM2	-1.930533	1.06E-09	3.51E-08
HIST1H2BF	-3.8599	6.27E-46	5.79E-43	TAS2R46	-1.561942	1.18E-09	3.87E-08
HIST1H3C	-4.424121	2.64E-44	2.31E-41	TRIM71	1.7211962	1.21E-09	3.93E-08
HIST1H3J	-3.906385	2.88E-44	2.40E-41	NPY1R	-1.121752	1.30E-09	4.19E-08
HIST2H2AC	-2.84128	8.01E-44	6.17E-41	ALDH1A2	-1.433192	1.34E-09	4.29E-08
HIST1H4B	-4.8358	8.09E-44	6.17E-41	EEF1A2	1.245248	1.60E-09	5.02E-08
HIST1H2AL	-4.186792	3.04E-43	2.22E-40	CDKL4	-1.097332	1.82E-09	5.58E-08
HIST2H2AB	-4.857422	3.34E-43	2.34E-40	RNF17	1.5188036	1.91E-09	5.81E-08
GPR83	3.1011893	9.82E-43	6.62E-40	PRLHR	-2.060906	2.08E-09	6.28E-08
HIST1H2BE	-3.790123	1.60E-42	1.01E-39	PRSS1	1.6241321	2.36E-09	6.95E-08
HIST1H2AB	-4.78184	1.62E-42	1.01E-39	KCNF1	1.0619567	2.50E-09	7.35E-08
HIST1H2BC	-2.675025	3.89E-40	2.35E-37	TBR1	1.6127779	2.52E-09	7.39E-08
HIST1H2BM	-4.7136	4.16E-38	2.43E-35	RIMS4	1.3862448	2.55E-09	7.43E-08
HIST1H2BG	-2.685197	7.51E-38	4.25E-35	DDIT4L	1.1306764	2.59E-09	7.47E-08
HIST2H2BF	-2.601902	2.90E-37	1.59E-34	BEST2	-1.350897	2.66E-09	7.63E-08
HIST1H3I	-4.158997	1.59E-35	8.46E-33	CTAG2	2.5860833	2.84E-09	8.10E-08
HIST1H4L	-5.438196	1.91E-34	9.87E-32	GP2	-1.96062	3.35E-09	9.38E-08
HIST1H2BB	-6.432312	3.78E-34	1.89E-31	SLC38A11	1.184307	3.36E-09	9.39E-08
HIST1H2BJ	-2.259606	4.84E-33	2.35E-30	ZMAT4	1.3585469	3.48E-09	9.61E-08
HIST1H2AG	-2.067482	6.50E-33	3.08E-30	DUSP9	1.1369299	3.52E-09	9.68E-08
HIST1H3G	-2.463551	1.54E-32	7.10E-30	SLIT1	1.0181144	3.57E-09	9.82E-08
HIST1H3D	-2.352117	1.78E-32	7.98E-30	CLPS	2.056425	3.75E-09	1.02E-07
ZNF460	-1.719692	1.62E-29	7.12E-27	CYP3A4	-1.265483	3.99E-09	1.07E-07
HIST1H4H	-2.333338	3.83E-29	1.64E-26	PLPPR3	1.2163644	4.26E-09	1.14E-07
REP15	-2.026697	6.44E-29	2.69E-26	FGF19	1.4561401	4.27E-09	1.14E-07
NNAT	-2.201727	7.88E-28	3.21E-25	TTC29	-2.282844	4.72E-09	1.24E-07
HIST1H2AE	-2.066772	1.02E-27	4.05E-25	SOHLH2	1.8638175	4.75E-09	1.25E-07

COLEC10	-2.007024	2.37E-27	9.22E-25	TMEM249	1.0182247	4.78E-09	1.25E-07
HIST2H3D	-2.538414	3.14E-27	1.20E-24	ZPBP2	1.4614946	5.20E-09	1.33E-07
TFAP2B	4.9324	1.16E-26	4.34E-24	HCN4	1.2190824	5.24E-09	1.34E-07
LHX3	3.4401398	2.92E-26	1.07E-23	TRPM3	1.3254694	5.98E-09	1.50E-07
CHRNA4	3.7212652	4.13E-26	1.48E-23	GAL	1.0286609	6.20E-09	1.55E-07
GPR22	-2.385373	1.03E-25	3.55E-23	CTRC	1.0305868	6.49E-09	1.60E-07
HIST1H2BN	-1.581651	1.11E-25	3.75E-23	PHF21B	1.3754089	6.67E-09	1.64E-07
CLDN6	2.6151865	1.57E-25	5.17E-23	CLDN19	1.482786	6.86E-09	1.67E-07
PNMT	2.2610117	1.59E-25	5.17E-23	C19orf81	1.3266556	7.12E-09	1.73E-07
HIST1H2BH	-1.934131	1.66E-24	5.20E-22	DNASE1L3	-1.032325	7.32E-09	1.76E-07
HIST1H4A	-3.340588	2.02E-24	6.21E-22	MAEL	-1.418633	7.40E-09	1.78E-07
HIST4H4	-1.544525	3.77E-24	1.14E-21	GPR52	-1.554192	7.54E-09	1.81E-07
CALCB	3.9414688	1.80E-23	5.33E-21	PSG9	-2.634426	8.13E-09	1.94E-07
UGT2B4	-3.686344	1.82E-23	5.33E-21	CLDN8	-1.885228	9.01E-09	2.13E-07
CPA1	3.6696948	2.13E-23	6.11E-21	KIRREL2	1.0857926	9.07E-09	2.14E-07
ZBTB20	-1.331365	6.45E-23	1.82E-20	MROH2B	1.8297208	9.07E-09	2.14E-07
TCAP	1.5751864	9.26E-23	2.58E-20	AMER3	1.5845629	1.10E-08	2.52E-07
IGDCC3	2.7507925	1.05E-22	2.88E-20	TAS2R43	-1.382288	1.11E-08	2.54E-07
DCAF4L2	4.568086	1.48E-22	4.00E-20	FAM153A	-1.073024	1.49E-08	3.26E-07
CRCT1	-4.279701	2.93E-22	7.77E-20	C1QL4	1.1601898	1.56E-08	3.37E-07
HIST1H1C	-1.428279	8.81E-22	2.31E-19	DEFA6	-1.68007	1.57E-08	3.40E-07
TRPA1	-1.350696	9.51E-22	2.45E-19	CNTNAP4	1.700302	1.67E-08	3.56E-07
UGT3A1	3.6949494	9.66E-22	2.45E-19	LRRTM1	1.4596784	1.89E-08	3.97E-07
SYNGR4	-2.051495	1.44E-21	3.60E-19	CCDC190	-1.856342	1.93E-08	4.05E-07
SFTPC	4.635231	1.46E-21	3.60E-19	MYT1L	1.3585142	1.97E-08	4.10E-07
KCNQ2	2.632427	1.71E-21	4.17E-19	MMP13	-1.156005	1.97E-08	4.11E-07
FSIP2	-1.34768	1.92E-21	4.60E-19	C11orf21	1.0143236	2.06E-08	4.28E-07
HIST1H3H	-1.498006	2.13E-21	5.05E-19	C10orf113	-1.487899	2.13E-08	4.39E-07
CACNG6	2.9432425	2.74E-21	6.40E-19	LGALS14	1.7647053	2.25E-08	4.60E-07
LIPC	2.1783989	3.34E-21	7.71E-19	PCSK2	1.4591277	2.37E-08	4.83E-07
KRTAP4-6	3.6731871	5.67E-21	1.29E-18	CELF3	1.1027544	2.61E-08	5.27E-07
CLCA2	-2.57009	7.84E-21	1.76E-18	APOF	-1.491609	2.90E-08	5.77E-07
SELENOP	-1.184938	8.77E-21	1.93E-18	ITLN2	-1.633531	2.96E-08	5.85E-07
SLC16A7	-1.431203	8.81E-21	1.93E-18	UROC1	1.1232598	3.12E-08	6.11E-07
CXorf67	3.6258539	1.04E-20	2.25E-18	GPR15	-1.138453	3.24E-08	6.30E-07
HIST1H1A	-3.83254	1.59E-20	3.35E-18	KRT3	1.4234209	3.30E-08	6.40E-07
MYBPC3	-1.601179	1.66E-20	3.46E-18	GDF10	1.5681822	3.64E-08	6.97E-07
GPR50	3.2856688	1.87E-20	3.86E-18	RS1	1.1829526	3.66E-08	7.00E-07
AMBN	3.3534173	1.99E-20	4.06E-18	NTSR1	1.2325421	3.74E-08	7.12E-07
DPYSL5	2.3071154	2.15E-20	4.31E-18	CCKBR	1.630676	3.82E-08	7.24E-07
CLPSL1	3.4629815	2.16E-20	4.31E-18	FGF21	1.3169641	4.04E-08	7.60E-07
FCGBP	-1.826024	2.74E-20	5.40E-18	NTN3	1.0107566	4.24E-08	7.93E-07
GALP	3.6730518	3.97E-20	7.74E-18	CALB1	1.7198315	4.38E-08	8.14E-07
TAS2R30	-2.736361	5.14E-20	9.90E-18	SEMG2	1.9231293	4.64E-08	8.57E-07
BTBD18	-1.994763	5.78E-20	1.10E-17	BMP3	-1.105786	4.69E-08	8.63E-07
SOHLH1	3.5796366	8.12E-20	1.53E-17	IDO1	1.0517777	4.69E-08	8.63E-07

GALNTL6	-1.933532	1.09E-19	2.03E-17	OBP2A	1.2946658	4.76E-08	8.76E-07
CHRND	2.9554846	1.11E-19	2.05E-17	FAM216B	-1.038781	4.93E-08	9.03E-07
AIRE	2.3127612	1.33E-19	2.42E-17	SALL3	-2.469962	5.33E-08	9.65E-07
HIST1H4J	-1.66989	1.38E-19	2.50E-17	MAGEA10	2.1327507	5.36E-08	9.70E-07
CHGB	2.3815311	1.41E-19	2.52E-17	CELA3B	1.3429344	5.41E-08	9.78E-07
HRH3	3.0412941	1.94E-19	3.44E-17	KLK13	-1.290529	5.63E-08	1.01E-06
NEUROD4	3.6064562	2.07E-19	3.64E-17	ADGRA1	1.4171038	6.05E-08	1.08E-06
HIST1H4K	-1.787342	3.34E-19	5.80E-17	ATP6V0A4	1.2765261	6.34E-08	1.12E-06
FER1L6	-1.792425	5.30E-19	9.10E-17	RPRM	1.0351558	7.00E-08	1.22E-06
PRH2	-1.909778	5.98E-19	1.02E-16	AMY2A	1.6857865	7.03E-08	1.22E-06
CTRB2	2.6115442	1.28E-18	2.16E-16	CATSPER4	1.2242566	7.12E-08	1.23E-06
PNLIP	4.3309242	1.54E-18	2.57E-16	KRTAP1-1	1.5833409	7.72E-08	1.32E-06
WNT3A	1.9093129	1.87E-18	3.10E-16	AQP5	1.5785877	8.85E-08	1.49E-06
NXPE2	-1.383417	2.20E-18	3.61E-16	MEP1B	-1.00992	9.42E-08	1.56E-06
HEPACAM2	-1.781792	2.43E-18	3.94E-16	GABRP	1.2089313	9.53E-08	1.58E-06
CALCA	2.6355326	2.45E-18	3.94E-16	CHRNA9	-1.165317	1.28E-07	2.03E-06
SEZ6L	1.9910339	3.42E-18	5.41E-16	ZFR2	1.1164435	1.39E-07	2.18E-06
FOXR2	3.2552702	3.54E-18	5.53E-16	KLK6	1.1208537	1.42E-07	2.21E-06
CNBD2	1.3854824	3.77E-18	5.84E-16	KRT13	-1.327705	1.45E-07	2.24E-06
LRRTM2	-1.194066	3.89E-18	5.99E-16	DCT	1.4206236	1.47E-07	2.27E-06
POU5F2	-2.300261	3.99E-18	6.08E-16	ANXA8	-1.571167	1.62E-07	2.47E-06
CT45A1	3.9735267	4.24E-18	6.41E-16	UNC5D	-1.010811	1.69E-07	2.56E-06
HTR3C	-2.628374	5.32E-18	7.90E-16	RPRML	1.4929387	1.76E-07	2.65E-06
TUBA3C	3.0417264	6.73E-18	9.91E-16	CAMKV	1.1629299	1.76E-07	2.65E-06
ANO7	-1.241772	7.93E-18	1.16E-15	SULT1E1	1.3683322	1.90E-07	2.82E-06
KIF1A	1.9384169	1.07E-17	1.56E-15	NFE4	1.3070185	1.92E-07	2.84E-06
ADAD2	-2.24899	1.62E-17	2.33E-15	PRSS21	1.4879023	1.97E-07	2.90E-06
PPP1R17	3.0309667	1.78E-17	2.54E-15	FOXN4	1.2547342	2.05E-07	3.00E-06
NEUROD2	2.0425971	2.49E-17	3.49E-15	ZIC4	1.9091651	2.10E-07	3.05E-06
C16orf74	1.1924911	2.56E-17	3.57E-15	BBOX1	-1.030958	2.32E-07	3.33E-06
CHRNA2	2.5020757	3.63E-17	5.01E-15	FAM181B	-1.108728	2.36E-07	3.37E-06
NMRK2	3.1184518	3.66E-17	5.01E-15	CLEC2A	2.09194	2.46E-07	3.50E-06
KRT78	-3.076271	4.30E-17	5.85E-15	CES1	1.0343216	2.48E-07	3.52E-06
B3GNT6	-1.987156	4.48E-17	6.04E-15	IL17REL	-1.042948	2.90E-07	4.04E-06
LRRN3	-1.22144	5.39E-17	7.21E-15	PGC	1.1524551	2.96E-07	4.12E-06
SBK2	2.9255391	6.37E-17	8.46E-15	PRAME	1.1499546	2.97E-07	4.13E-06
ATP2B3	2.1469247	6.54E-17	8.62E-15	HIST2H3C	-1.197546	3.11E-07	4.29E-06
CTRB1	2.8400495	7.63E-17	9.90E-15	DPCR1	1.4130628	3.13E-07	4.31E-06
PTGER2	-1.078332	1.20E-16	1.55E-14	ERVW-1	-1.088463	3.18E-07	4.36E-06
KLHL32	-1.161251	1.28E-16	1.64E-14	KRT6C	-1.548101	3.30E-07	4.50E-06
UNCX	3.3110307	1.41E-16	1.79E-14	SAA2-SAA4	1.329412	3.76E-07	5.04E-06
CELA2A	2.3608436	1.47E-16	1.85E-14	CD200R1L	-1.259916	4.09E-07	5.42E-06
GRIP2	-1.202851	1.84E-16	2.31E-14	TF	1.0677624	4.15E-07	5.48E-06
SLC6A15	2.3014284	2.27E-16	2.82E-14	SH3GL3	1.3168084	4.21E-07	5.52E-06
NRAP	-1.695567	2.70E-16	3.31E-14	SAA2	1.0116294	4.33E-07	5.65E-06
RASD1	-1.241215	3.66E-16	4.45E-14	SCRT2	1.5906361	4.50E-07	5.83E-06

GALNT8	-1.370788	3.94E-16	4.77E-14	ATCAY	1.0095636	4.63E-07	5.97E-06
PNLDC1	1.635281	4.42E-16	5.27E-14	USP26	-1.208427	4.65E-07	5.98E-06
CRP	-2.604514	5.51E-16	6.53E-14	TMIGD1	-1.357469	5.20E-07	6.58E-06
RGS13	-1.462195	6.22E-16	7.32E-14	GOLGA6L2	1.3520623	5.26E-07	6.63E-06
C1orf61	1.5836229	1.24E-15	1.42E-13	KLF14	1.053364	5.48E-07	6.87E-06
FABP3	1.3122274	1.30E-15	1.48E-13	CRHR1	1.4227638	5.71E-07	7.13E-06
CACNG7	2.1056592	1.31E-15	1.48E-13	HCAR1	1.0489068	5.78E-07	7.19E-06
OBP2B	2.3972607	1.64E-15	1.85E-13	SLC38A3	1.1008015	6.69E-07	8.16E-06
ABCA4	-1.515138	1.73E-15	1.92E-13	FOXL2	1.3721146	7.15E-07	8.64E-06
MYL3	1.6484107	2.35E-15	2.58E-13	LRRC14B	1.0284391	7.23E-07	8.71E-06
PRSS56	3.0794769	2.41E-15	2.62E-13	LCN6	-1.033465	8.21E-07	9.74E-06
SLC6A3	1.6352569	3.12E-15	3.38E-13	SSX5	1.9028616	8.42E-07	9.97E-06
F7	1.7727683	3.39E-15	3.65E-13	VSTM2A	-1.170211	9.86E-07	1.14E-05
LRP1B	2.0017729	3.87E-15	4.12E-13	S100G	-1.215211	1.15E-06	1.30E-05
AQP3	-1.243876	5.55E-15	5.86E-13	PYY	-1.218362	1.18E-06	1.33E-05
MSMB	-2.801676	6.56E-15	6.81E-13	DCHS2	1.0566934	1.23E-06	1.38E-05
ST6GALNAC1	-1.00385	6.57E-15	6.81E-13	KRTAP3-1	1.3440443	1.25E-06	1.40E-05
COL20A1	1.8096008	6.83E-15	7.04E-13	HYDIN	1.0003167	1.29E-06	1.43E-05
C16orf54	-1.097119	6.92E-15	7.09E-13	DLX3	1.1948495	1.31E-06	1.45E-05
HPR	-2.093661	7.51E-15	7.65E-13	LYPD2	1.570215	1.33E-06	1.46E-05
IFNK	-2.645259	8.75E-15	8.86E-13	ZSCAN10	1.0206213	1.42E-06	1.55E-05
COX8C	2.3314199	1.28E-14	1.27E-12	OTOP2	-1.411608	1.43E-06	1.55E-05
LMX1A	2.0590215	1.35E-14	1.34E-12	INHA	1.1093779	1.49E-06	1.60E-05
PPBP	2.7211255	1.87E-14	1.81E-12	SH3GL2	1.0860603	1.50E-06	1.61E-05
CBFA2T3	-1.232814	1.87E-14	1.81E-12	CNGA3	-1.160729	1.50E-06	1.61E-05
ALOX15B	-1.34711	1.91E-14	1.84E-12	OR1N2	-2.122451	1.51E-06	1.62E-05
FBXO2	1.3652382	2.00E-14	1.91E-12	CYP2W1	1.0473038	1.62E-06	1.70E-05
SEZ6	1.8076619	2.07E-14	1.97E-12	COL2A1	1.3105674	1.63E-06	1.71E-05
NEURL1	-1.214889	2.19E-14	2.07E-12	FTCD	1.0838418	1.71E-06	1.78E-05
SHISA7	1.7365255	2.24E-14	2.12E-12	PRB2	-1.279735	1.72E-06	1.79E-05
HIST1H4F	-3.418083	2.63E-14	2.46E-12	ZFP42	1.5266873	1.78E-06	1.84E-05
AKAIN1	2.1412708	3.30E-14	3.06E-12	NKX2-5	1.3882698	1.84E-06	1.89E-05
UTS2B	1.6061676	3.90E-14	3.56E-12	REG4	-1.181563	1.85E-06	1.91E-05
SLC38A8	2.0291611	4.39E-14	3.97E-12	MAGEC1	1.9262589	1.93E-06	1.97E-05
ARHGAP36	2.1583854	4.58E-14	4.09E-12	TYR	1.6209184	1.98E-06	2.01E-05
P2RY4	-1.44199	5.04E-14	4.48E-12	SLC7A14	1.1815676	2.11E-06	2.12E-05
RBM44	-1.013009	6.49E-14	5.71E-12	PCDHB8	-1.024812	2.31E-06	2.28E-05
SCG3	1.7096702	9.83E-14	8.53E-12	AP3B2	1.053461	2.31E-06	2.28E-05
TPSG1	-1.33046	1.02E-13	8.77E-12	INSL5	-1.515049	2.47E-06	2.41E-05
TCHH	1.5514011	1.16E-13	9.89E-12	CT45A10	2.0532015	2.48E-06	2.41E-05
CHST5	-1.346512	1.56E-13	1.32E-11	PRMT8	1.0860917	2.48E-06	2.42E-05
CHST8	1.7661954	1.86E-13	1.56E-11	B4GALNT2	-1.194658	2.52E-06	2.45E-05
CAPN9	-1.244766	2.01E-13	1.68E-11	SLC17A8	-1.183282	2.59E-06	2.50E-05
SFTPA1	2.8402856	2.18E-13	1.81E-11	SYT4	1.1869242	2.64E-06	2.55E-05
TAS2R13	-1.970174	2.49E-13	2.03E-11	TLX3	1.4774075	2.95E-06	2.80E-05
LINC00694	-1.186399	2.50E-13	2.03E-11	KCNE1B	-1.742779	3.08E-06	2.90E-05

SI	-1.642304	2.72E-13	2.19E-11	UGT2B17	-1.145653	3.19E-06	2.98E-05
KIF19	-1.474135	2.99E-13	2.38E-11	SEC14L4	1.1194448	3.23E-06	3.01E-05
DSG1	-1.081652	3.07E-13	2.44E-11	DMRT3	1.0032422	3.23E-06	3.01E-05
SPRR2B	-2.963448	3.10E-13	2.45E-11	REG3G	1.7782947	3.46E-06	3.18E-05
NAT8L	1.3652195	3.22E-13	2.53E-11	CDC20B	1.0937419	3.75E-06	3.40E-05
SERPINB13	-3.50036	3.31E-13	2.59E-11	OVCH1	-1.143234	3.92E-06	3.54E-05
CCDC83	-1.987447	3.59E-13	2.77E-11	RLBP1	1.0538574	4.70E-06	4.14E-05
GLYATL2	-1.521598	3.81E-13	2.93E-11	VSTM2B	1.497234	4.88E-06	4.28E-05
KCNJ13	-1.995785	4.94E-13	3.75E-11	SAA4	1.0528389	4.89E-06	4.29E-05
SLITRK1	2.1966833	5.27E-13	3.98E-11	SLC36A2	1.1487226	4.92E-06	4.30E-05
ATOH1	-1.351333	6.71E-13	5.00E-11	DLK1	1.6953771	5.06E-06	4.41E-05
TMPRSS11D	-2.144702	7.00E-13	5.20E-11	CPA2	1.0129871	5.18E-06	4.50E-05
SLC4A4	-1.272903	8.90E-13	6.47E-11	AHSG	1.3056197	5.29E-06	4.57E-05
FGF6	2.5815135	8.97E-13	6.50E-11	AQP4	1.2052366	5.50E-06	4.73E-05
FGL1	-2.400341	9.23E-13	6.63E-11	LKAAEAR1	1.2233895	5.51E-06	4.73E-05
BTN1A1	1.397147	9.26E-13	6.63E-11	BEST3	1.1062867	5.79E-06	4.93E-05
NR0B1	1.8765318	1.01E-12	7.13E-11	FZD10	1.0670724	6.33E-06	5.31E-05
TMEM61	-1.080952	1.14E-12	7.91E-11	GUCA2B	-1.134006	6.92E-06	5.74E-05
ALK	-1.825641	1.16E-12	8.06E-11	AFP	1.1502473	7.32E-06	6.02E-05
SLC8A3	-1.376045	1.23E-12	8.49E-11	CELA3A	1.2043448	7.45E-06	6.11E-05
MAGEB1	2.7578546	1.33E-12	9.11E-11	ACTL6B	1.1052772	7.52E-06	6.16E-05
IRGM	-2.019513	1.46E-12	9.91E-11	KRT4	1.2067244	7.58E-06	6.21E-05
ZDHHC22	1.9236601	1.54E-12	1.04E-10	SCGB3A2	-1.285184	8.06E-06	6.55E-05
PRSS48	-1.771238	1.59E-12	1.07E-10	KRTAP13-2	-1.479632	8.53E-06	6.86E-05
CYP1A1	-2.42666	1.93E-12	1.27E-10	SFTPB	1.2834915	9.11E-06	7.23E-05
VSIG1	-1.443703	2.07E-12	1.36E-10	SULT2A1	1.1996299	9.23E-06	7.31E-05
LUZP2	-1.108808	2.18E-12	1.42E-10	KRT5	-1.437554	9.31E-06	7.36E-05
GSTA1	-1.385475	2.28E-12	1.48E-10	SORCS3	1.1064193	9.54E-06	7.51E-05
DLX5	1.4425714	2.46E-12	1.58E-10	LIPF	1.6156862	1.21E-05	9.25E-05
FAM177B	-1.345795	2.54E-12	1.62E-10	PBOV1	-1.268291	1.36E-05	0.0001016
ZNF479	-3.394555	2.60E-12	1.66E-10	NOTUM	1.0501836	1.37E-05	0.0001019
CRNN	-2.84563	2.91E-12	1.83E-10	SLITRK6	-1.014594	1.38E-05	0.0001024
SAA1	1.4759248	3.29E-12	2.04E-10	HBZ	1.876635	1.48E-05	0.0001094
KRT31	2.3560733	4.23E-12	2.59E-10	RPTN	1.5522914	1.51E-05	0.0001109
ZNF578	-1.263417	6.75E-12	3.97E-10	LINC01314	1.0128324	1.53E-05	0.0001124
COL26A1	1.1852709	7.29E-12	4.24E-10	KLRG2	1.060652	1.55E-05	0.0001137
RHBDL3	-1.289823	8.15E-12	4.71E-10	TKTL1	1.464721	1.72E-05	0.0001239
RASL10B	1.0540383	8.28E-12	4.78E-10	ANKRD34C	1.2183323	1.72E-05	0.000124
TMEM213	1.7880091	8.87E-12	5.07E-10	ATP12A	-1.336882	1.77E-05	0.0001267
TAAR1	-2.817762	9.89E-12	5.61E-10	AC011473.4	-1.641567	1.83E-05	0.0001306
HTR3E	-1.611537	1.07E-11	6.03E-10	LRRC53	1.3376302	1.96E-05	0.0001382
KLK4	1.5065618	1.10E-11	6.18E-10	GFY	1.100956	1.97E-05	0.0001388
KRT37	1.8820649	1.10E-11	6.18E-10	GNAT3	-1.517338	2.05E-05	0.0001431
ITLN1	-1.700398	1.12E-11	6.28E-10	BHLHA9	1.1383672	2.07E-05	0.000144
C20orf204	1.1371742	1.18E-11	6.57E-10	SCGB1A1	-1.739115	2.23E-05	0.0001542
HSF5	1.6137511	1.31E-11	7.17E-10	IGFALS	-1.009595	2.42E-05	0.000165

CER1	2.0783045	1.34E-11	7.30E-10	MYBPHL	1.1142612	3.10E-05	0.000203
RHBG	1.2076119	1.38E-11	7.52E-10	GPHA2	1.0048822	3.12E-05	0.0002036
EPHB6	1.4238447	1.39E-11	7.57E-10	KRT32	1.250768	3.12E-05	0.0002036
IGF2	2.0018263	1.58E-11	8.55E-10	L1TD1	-1.003548	3.15E-05	0.0002052
PAGE2	2.01334	1.77E-11	9.49E-10	TRPM1	1.0784805	3.27E-05	0.0002117
CRISP3	-2.553675	1.86E-11	9.95E-10	SPATA22	-1.081709	3.86E-05	0.0002425
SLC1A6	1.8222299	1.94E-11	1.03E-09	BRDT	1.0092331	4.10E-05	0.0002552
CLCA4	-1.759103	2.09E-11	1.10E-09	CA1	-1.095544	4.13E-05	0.0002569
SLC26A9	-1.89822	2.36E-11	1.24E-09	GCG	-1.365199	4.21E-05	0.0002609
TSPAN32	1.198575	2.50E-11	1.31E-09	CPN1	1.0770569	4.38E-05	0.0002699
TMCO2	-1.837882	2.52E-11	1.31E-09	GALNT13	-1.017826	4.40E-05	0.0002705
SPRR2E	-2.893625	3.64E-11	1.83E-09	MYOC	1.1785249	4.99E-05	0.0003002
PRODH2	2.1081067	3.96E-11	1.99E-09	XAGE2	1.6363979	5.08E-05	0.0003043
TPH1	-1.121172	4.00E-11	2.00E-09	MS4A12	-1.041735	6.05E-05	0.0003524
LYPD8	-1.255869	4.73E-11	2.35E-09	FAM25A	1.2068566	6.91E-05	0.0003919
CPLX2	1.3708984	4.86E-11	2.40E-09	PAX4	1.2211239	6.98E-05	0.0003958
CCDC60	-1.494497	5.00E-11	2.47E-09	DRD5	-1.210499	7.13E-05	0.0004033
EMX2	2.0804659	5.04E-11	2.48E-09	HSD3B2	-1.109224	7.54E-05	0.0004235
TMEM210	-1.619843	5.12E-11	2.51E-09	IVL	-1.521663	7.75E-05	0.000434
NCAN	1.6390064	5.66E-11	2.77E-09	SOAT2	1.0053323	8.79E-05	0.0004855
OGDHL	1.2791147	5.80E-11	2.82E-09	MAGEB17	1.0668633	9.38E-05	0.0005127
TP63	-1.288461	6.36E-11	3.07E-09	MUC15	-1.302314	9.47E-05	0.000516
CDH22	1.9058161	6.56E-11	3.15E-09	PRR9	1.4250586	0.0001108	0.0005905
CLCA1	-1.794184	6.62E-11	3.17E-09	HSPB3	1.0249806	0.0001234	0.0006434
SCGB2A1	-1.223694	6.68E-11	3.19E-09	PIP	-1.161274	0.0001277	0.0006615
VSIG2	-1.22858	7.10E-11	3.38E-09	PROK1	1.1172041	0.0001768	0.0008693
CHST13	1.0559363	7.12E-11	3.38E-09	BPIFB2	1.2936928	0.0001803	0.0008836
KRT14	-2.600927	7.86E-11	3.69E-09	LIN28A	1.2498911	0.0001821	0.0008916
PDYN	2.3990366	7.88E-11	3.69E-09	SERPINA9	-1.09358	0.0001918	0.0009308
PCDHA1	-1.888699	8.61E-11	3.98E-09	ZIC1	1.2704971	0.0002008	0.000968
ZG16	-1.709932	8.70E-11	4.01E-09	TCP11	-1.086815	0.0002144	0.001023
TMEM229A	2.1742857	8.76E-11	4.02E-09	SLC18A3	1.0048659	0.0002245	0.0010626
DGKB	-1.595286	8.83E-11	4.03E-09	KRT24	-1.367046	0.0002348	0.0011019
MUC21	1.9495458	9.00E-11	4.09E-09	DMRT1	1.1719634	0.0002852	0.0012999
KRT38	1.9695272	1.12E-10	5.01E-09	NDST4	1.0872445	0.0003276	0.0014613
TAS2R50	-1.649773	1.16E-10	5.14E-09	LIN28B	1.5407586	0.0003332	0.001483
GNG13	-1.36269	1.24E-10	5.46E-09	NKX6-3	1.3317919	0.0003625	0.0015848
IL36G	-1.31316	1.24E-10	5.46E-09	TRDN	-1.065055	0.0004016	0.0017268
RPE65	1.7464619	1.40E-10	6.09E-09	CCL25	1.1719039	0.0004243	0.0018047
IRX4	2.0885753	1.64E-10	7.07E-09	GC	-1.184243	0.000529	0.0021671
MIA	1.3723102	1.74E-10	7.41E-09	SLURP1	-1.017284	0.0005527	0.0022543
TCN1	-1.540012	1.79E-10	7.63E-09	APOB	1.0135529	0.0005925	0.0023872
SHISA3	-1.096602	1.88E-10	7.95E-09	PTF1A	1.1816283	0.0006012	0.0024161
IL1RAPL2	-2.270044	1.92E-10	8.09E-09	DEFA5	-1.094099	0.0008245	0.0031474
JCHAIN	-1.222295	2.02E-10	8.47E-09	KRT35	1.0278618	0.0008331	0.0031727
SLC7A10	1.6061239	2.07E-10	8.64E-09	GAD2	-1.409394	0.0008885	0.0033676

MGAT5B	1.0360896	2.22E-10	9.15E-09	MT4	1.3200531	0.0010781	0.0039863
CDH12	2.2596061	2.58E-10	1.04E-08	NTS	-1.050255	0.0010913	0.0040299
NEUROD1	2.2463322	2.69E-10	1.07E-08	RHAG	1.2123393	0.0011909	0.0043392
CHRNA7	-1.244649	2.69E-10	1.07E-08	SAGE1	1.2292124	0.0013334	0.0047744
GLP1R	1.5805223	2.77E-10	1.09E-08	XKR7	1.0130495	0.0015198	0.0053272
POU3F2	1.2794791	2.84E-10	1.11E-08	BPIFB4	-1.098732	0.0016908	0.0058347
MLN	2.289581	2.86E-10	1.12E-08	HOXC13	1.0902752	0.0019278	0.0065342
NTRK3	1.177568	2.94E-10	1.14E-08	DAZ1	1.2895124	0.0031927	0.0099007
MMP8	1.6981865	3.32E-10	1.27E-08	SP9	1.0371283	0.0033135	0.0102245
PLSCR5	-2.674294	3.49E-10	1.33E-08	SSX1	-1.267772	0.0039466	0.0118523
CALML5	2.3122375	3.88E-10	1.47E-08	MAGEA9B	1.2028866	0.0047881	0.0139105
CTNNA2	1.7490958	3.95E-10	1.50E-08	HOXC12	1.0284406	0.0058428	0.0164484
COX7B2	2.4519109	4.03E-10	1.51E-08	CDH9	1.0418542	0.0063574	0.0176225
AC007040.2	1.1692465	4.03E-10	1.51E-08	HBE1	1.0580499	0.0066571	0.0183057
RGN	1.1409741	4.38E-10	1.63E-08	MAGEB2	-1.153926	0.0085544	0.0225164
IGF2BP1	1.6784141	4.39E-10	1.63E-08				

Table S8. Functional enrichment of DEG between high- and low-STXBP5-AS1 expression groups.

Category	Term	Count	%	P Value
KEGG_PATHWAY	hsa05322:Systemic lupus erythematosus	47	0.079195	3.79E-36
KEGG_PATHWAY	hsa05034:Alcoholism	51	0.085935	1.21E-34
KEGG_PATHWAY	hsa05203:Viral carcinogenesis	25	0.042125	3.33E-08
KEGG_PATHWAY	hsa04972:Pancreatic secretion	12	0.02022	1.83E-04
KEGG_PATHWAY	hsa04080:Neuroactive ligand-receptor interaction	21	0.035385	5.86E-04
KEGG_PATHWAY	hsa04974:Protein digestion and absorption	10	0.01685	0.002013
KEGG_PATHWAY	hsa05202:Transcriptional misregulation in cancer	13	0.021905	0.007588
KEGG_PATHWAY	hsa04742:Taste transduction	6	0.01011	0.014303
KEGG_PATHWAY	hsa00512:Mucin type O-Glycan biosynthesis	5	0.008425	0.016768
KEGG_PATHWAY	hsa04975:Fat digestion and absorption	5	0.008425	0.03593
KEGG_PATHWAY	hsa00140:Steroid hormone biosynthesis	6	0.01011	0.0384
KEGG_PATHWAY	hsa05033:Nicotine addiction	5	0.008425	0.038947
KEGG_PATHWAY	hsa05204:Chemical carcinogenesis	7	0.011795	0.043855
GOTERM_CC_DIRECT	GO:0000786~nucleosome	50	0.08425	7.71E-50
GOTERM_BP_DIRECT	GO:0006334~nucleosome assembly	41	0.069085	5.25E-32
GOTERM_BP_DIRECT	GO:0032200~telomere organization	20	0.0337	7.36E-24
GOTERM_CC_DIRECT	GO:0005576~extracellular region	124	0.208941	2.32E-23
GOTERM_BP_DIRECT	GO:0000183~chromatin silencing at rDNA	22	0.03707	2.64E-23
GOTERM_CC_DIRECT	GO:0000788~nuclear nucleosome	23	0.038755	1.90E-22
GOTERM_MF_DIRECT	GO:0046982~protein heterodimerization activity	60	0.1011	3.70E-22
GOTERM_BP_DIRECT	GO:0006335~DNA replication-dependent nucleosome assembly	20	0.0337	1.00E-21
GOTERM_BP_DIRECT	GO:0045814~negative regulation of gene expression, epigenetic	22	0.03707	9.57E-20
GOTERM_BP_DIRECT	GO:0044267~cellular protein metabolic process	30	0.05055	2.88E-19
GOTERM_BP_DIRECT	GO:0051290~protein heterotetramerization	20	0.0337	9.70E-19
GOTERM_BP_DIRECT	GO:0045815~positive regulation of gene expression, epigenetic	22	0.03707	1.87E-17
GOTERM_CC_DIRECT	GO:0000228~nuclear chromosome	20	0.0337	3.19E-16
GOTERM_MF_DIRECT	GO:0042393~histone binding	25	0.042125	7.75E-14
GOTERM_CC_DIRECT	GO:0005615~extracellular space	92	0.15502	8.68E-14
GOTERM_BP_DIRECT	GO:0031047~gene silencing by RNA	23	0.038755	7.78E-13
GOTERM_BP_DIRECT	GO:0045653~negative regulation of megakaryocyte differentiation	11	0.018535	1.34E-11
GOTERM_BP_DIRECT	GO:0060968~regulation of gene silencing	9	0.015165	7.11E-11
GOTERM_BP_DIRECT	GO:0016233~telomere capping	11	0.018535	3.07E-10
GOTERM_BP_DIRECT	GO:0006336~DNA replication-independent nucleosome assembly	11	0.018535	1.32E-09

GOTERM_CC_DIRECT	GO:0000784~nuclear chromosome, telomeric region	21	0.035385	1.66E-09
GOTERM_BP_DIRECT	GO:0006352~DNA-templated transcription, initiation	11	0.018535	4.86E-08
GOTERM_BP_DIRECT	GO:0007586~digestion	13	0.021905	2.11E-07
GOTERM_BP_DIRECT	GO:0034080~CENP-A containing nucleosome assembly	11	0.018535	3.06E-07
GOTERM_BP_DIRECT	GO:1904837~beta-catenin-TCF complex assembly	11	0.018535	3.06E-07
GOTERM_BP_DIRECT	GO:0019731~antibacterial humoral response	11	0.018535	3.86E-07
GOTERM_MF_DIRECT	GO:0042166~acetylcholine binding	9	0.015165	4.48E-07
GOTERM_BP_DIRECT	GO:0006342~chromatin silencing	11	0.018535	4.83E-07
GOTERM_MF_DIRECT	GO:0031492~nucleosomal DNA binding	11	0.018535	5.84E-07
GOTERM_MF_DIRECT	GO:0005198~structural molecule activity	24	0.04044	8.32E-07
GOTERM_MF_DIRECT	GO:0004889~acetylcholine-activated cation-selective channel activity	8	0.01348	2.61E-06
GOTERM_CC_DIRECT	GO:0005892~acetylcholine-gated channel complex	8	0.01348	4.41E-06
GOTERM_MF_DIRECT	GO:0004252~serine-type endopeptidase activity	23	0.038755	5.06E-06
GOTERM_BP_DIRECT	GO:0007271~synaptic transmission, cholinergic	9	0.015165	8.57E-06
GOTERM_BP_DIRECT	GO:0006303~double-strand break repair via nonhomologous end joining	11	0.018535	1.21E-05
GOTERM_MF_DIRECT	GO:0005179~hormone activity	13	0.021905	1.62E-05
GOTERM_CC_DIRECT	GO:0005882~intermediate filament	14	0.02359	3.13E-05
GOTERM_MF_DIRECT	GO:0015464~acetylcholine receptor activity	7	0.011795	3.71E-05
GOTERM_MF_DIRECT	GO:0015276~ligand-gated ion channel activity	8	0.01348	6.43E-05
GOTERM_CC_DIRECT	GO:0070062~extracellular exosome	118	0.198831	6.97E-05
GOTERM_CC_DIRECT	GO:0045095~keratin filament	12	0.02022	1.89E-04
GOTERM_CC_DIRECT	GO:0000790~nuclear chromatin	17	0.028645	2.21E-04
GOTERM_BP_DIRECT	GO:0035881~amacrine cell differentiation	4	0.00674	2.31E-04
GOTERM_BP_DIRECT	GO:0009235~cobalamin metabolic process	6	0.01011	2.76E-04
GOTERM_CC_DIRECT	GO:0034774~secretory granule lumen	5	0.008425	3.17E-04
GOTERM_BP_DIRECT	GO:0006508~proteolysis	30	0.05055	3.18E-04
GOTERM_BP_DIRECT	GO:0035094~response to nicotine	7	0.011795	6.18E-04
GOTERM_BP_DIRECT	GO:0002227~innate immune response in mucosa	6	0.01011	6.54E-04
GOTERM_BP_DIRECT	GO:0007626~locomotory behavior	10	0.01685	7.11E-04
GOTERM_BP_DIRECT	GO:0007631~feeding behavior	7	0.011795	7.16E-04
GOTERM_BP_DIRECT	GO:0008544~epidermis development	10	0.01685	7.75E-04
GOTERM_BP_DIRECT	GO:0006953~acute-phase response	7	0.011795	8.26E-04
GOTERM_BP_DIRECT	GO:0006811~ion transport	12	0.02022	0.001159
GOTERM_BP_DIRECT	GO:0007612~learning	8	0.01348	0.001233

GOTERM_CC_DIRECT	GO:0031012~extracellular matrix	20	0.0337	0.001454
GOTERM_MF_DIRECT	GO:0015171~amino acid transmembrane transporter activity	7	0.011795	0.002201
GOTERM_CC_DIRECT	GO:0001533~cornified envelope	7	0.011795	0.002296
GOTERM_MF_DIRECT	GO:0005230~extracellular ligand-gated ion channel activity	6	0.01011	0.002383
GOTERM_BP_DIRECT	GO:0010737~protein kinase A signaling	4	0.00674	0.002484
GOTERM_BP_DIRECT	GO:0098655~cation transmembrane transport	7	0.011795	0.002495
GOTERM_BP_DIRECT	GO:0007218~neuropeptide signaling pathway	10	0.01685	0.002622
GOTERM_BP_DIRECT	GO:0007596~blood coagulation	14	0.02359	0.002761
GOTERM_BP_DIRECT	GO:0007585~respiratory gaseous exchange	6	0.01011	0.002767
GOTERM_BP_DIRECT	GO:0050830~defense response to Gram-positive bacterium	9	0.015165	0.003184
GOTERM_BP_DIRECT	GO:0010842~retina layer formation	5	0.008425	0.003349
GOTERM_CC_DIRECT	GO:0034364~high-density lipoprotein particle	5	0.008425	0.003696
GOTERM_MF_DIRECT	GO:0030246~carbohydrate binding	14	0.02359	0.004602
GOTERM_MF_DIRECT	GO:0001664~G-protein coupled receptor binding	7	0.011795	0.0049
GOTERM_CC_DIRECT	GO:0005887~integral component of plasma membrane	60	0.1011	0.005193
GOTERM_MF_DIRECT	GO:0033038~bitter taste receptor activity	5	0.008425	0.005348
GOTERM_BP_DIRECT	GO:0001580~detection of chemical stimulus involved in sensory perception of bitter taste	6	0.01011	0.005678
GOTERM_BP_DIRECT	GO:0042391~regulation of membrane potential	8	0.01348	0.005936
GOTERM_CC_DIRECT	GO:0097440~apical dendrite	4	0.00674	0.005989
GOTERM_CC_DIRECT	GO:0043234~protein complex	23	0.038755	0.006172
GOTERM_BP_DIRECT	GO:0006810~transport	20	0.0337	0.006308
GOTERM_MF_DIRECT	GO:0008083~growth factor activity	12	0.02022	0.007416
GOTERM_MF_DIRECT	GO:0005499~vitamin D binding	3	0.005055	0.007839
GOTERM_BP_DIRECT	GO:0032225~regulation of synaptic transmission, dopaminergic	3	0.005055	0.007888
GOTERM_BP_DIRECT	GO:0007417~central nervous system development	10	0.01685	0.008137
GOTERM_BP_DIRECT	GO:0034220~ion transmembrane transport	14	0.02359	0.008315
GOTERM_BP_DIRECT	GO:0007613~memory	7	0.011795	0.008914
GOTERM_BP_DIRECT	GO:0050909~sensory perception of taste	5	0.008425	0.009274
GOTERM_MF_DIRECT	GO:0008236~serine-type peptidase activity	7	0.011795	0.009476
GOTERM_MF_DIRECT	GO:0005509~calcium ion binding	33	0.055605	0.009902
GOTERM_MF_DIRECT	GO:0005229~intracellular calcium activated chloride channel activity	4	0.00674	0.0101
GOTERM_MF_DIRECT	GO:0005184~neuropeptide hormone	5	0.008425	0.010347

	activity			
GOTERM_BP_DIRECT	GO:0098532~histone H3-K27 trimethylation	3	0.005055	0.011607
GOTERM_MF_DIRECT	GO:0003677~DNA binding	65	0.109525	0.011666
GOTERM_BP_DIRECT	GO:0018149~peptide cross-linking	6	0.01011	0.014458
GOTERM_BP_DIRECT	GO:0006776~vitamin A metabolic process	3	0.005055	0.01594
GOTERM_BP_DIRECT	GO:0042472~inner ear morphogenesis	6	0.01011	0.016926
GOTERM_MF_DIRECT	GO:0019904~protein domain specific binding	13	0.021905	0.017825
GOTERM_BP_DIRECT	GO:1902476~chloride transmembrane transport	8	0.01348	0.018249
GOTERM_BP_DIRECT	GO:0007010~cytoskeleton organization	11	0.018535	0.018873
GOTERM_CC_DIRECT	GO:0016323~basolateral plasma membrane	12	0.02022	0.019033
GOTERM_MF_DIRECT	GO:0005254~chloride channel activity	6	0.01011	0.019415
GOTERM_CC_DIRECT	GO:0005581~collagen trimer	8	0.01348	0.01978
GOTERM_BP_DIRECT	GO:0016584~nucleosome positioning	3	0.005055	0.02085
GOTERM_MF_DIRECT	GO:0008146~sulfotransferase activity	5	0.008425	0.021305
GOTERM_BP_DIRECT	GO:0035902~response to immobilization stress	4	0.00674	0.021751
GOTERM_BP_DIRECT	GO:0032094~response to food	4	0.00674	0.021751
GOTERM_CC_DIRECT	GO:0042599~lamellar body	3	0.005055	0.021949
GOTERM_BP_DIRECT	GO:0070588~calcium ion transmembrane transport	9	0.015165	0.022334
GOTERM_BP_DIRECT	GO:0030216~keratinocyte differentiation	7	0.011795	0.022672
GOTERM_CC_DIRECT	GO:0005796~Golgi lumen	8	0.01348	0.024354
GOTERM_MF_DIRECT	GO:0005104~fibroblast growth factor receptor binding	4	0.00674	0.024451
GOTERM_MF_DIRECT	GO:0031490~chromatin DNA binding	6	0.01011	0.02564
GOTERM_MF_DIRECT	GO:0004983~neuropeptide Y receptor activity	3	0.005055	0.026141
GOTERM_BP_DIRECT	GO:0072488~ammonium transmembrane transport	3	0.005055	0.026299
GOTERM_BP_DIRECT	GO:0035095~behavioral response to nicotine	3	0.005055	0.026299
GOTERM_BP_DIRECT	GO:0006814~sodium ion transport	7	0.011795	0.029884
GOTERM_BP_DIRECT	GO:0008306~associative learning	4	0.00674	0.031062
GOTERM_BP_DIRECT	GO:0001523~retinoid metabolic process	6	0.01011	0.031432
GOTERM_MF_DIRECT	GO:0035240~dopamine binding	3	0.005055	0.032061
GOTERM_BP_DIRECT	GO:0042359~vitamin D metabolic process	3	0.005055	0.032253
GOTERM_BP_DIRECT	GO:0051965~positive regulation of synapse assembly	6	0.01011	0.033407
GOTERM_BP_DIRECT	GO:0019433~triglyceride catabolic process	4	0.00674	0.03456
GOTERM_BP_DIRECT	GO:0008202~steroid metabolic process	5	0.008425	0.035174
GOTERM_BP_DIRECT	GO:0015701~bicarbonate transport	5	0.008425	0.037839
GOTERM_BP_DIRECT	GO:0007274~neuromuscular synaptic transmission	4	0.00674	0.038252

GOTERM_BP_DIRECT	GO:0007399~nervous system development	15	0.025275	0.04038
GOTERM_MF_DIRECT	GO:0042056~chemoattractant activity	4	0.00674	0.0418
GOTERM_MF_DIRECT	GO:0015254~glycerol channel activity	3	0.005055	0.045275
GOTERM_BP_DIRECT	GO:0021846~cell proliferation in forebrain	3	0.005055	0.045541
GOTERM_BP_DIRECT	GO:0001501~skeletal system development	9	0.015165	0.045808
GOTERM_MF_DIRECT	GO:0043565~sequence-specific DNA binding	23	0.038755	0.046034
GOTERM_BP_DIRECT	GO:0051781~positive regulation of cell division	5	0.008425	0.046508
GOTERM_BP_DIRECT	GO:0008203~cholesterol metabolic process	6	0.01011	0.046862
GOTERM_BP_DIRECT	GO:0031424~keratinization	5	0.008425	0.049623

Table S9. Survival analysis of DEG between high- and low-STXBP5-AS1 expression groups (Only gene list with P<0.05 is shown).

ID	P value	HR	Low 95%CI	High 95%CI
COX8C	0.002984	1.89384	1.24246	2.886715
KRT3	0.003443	1.890205	1.233775	2.89589
COLEC10	0.00769	0.561302	0.367089	0.858267
LUZP2	0.008279	0.563532	0.368157	0.862591
B3GNT6	0.008328	0.561804	0.366072	0.86219
CLCA1	0.011671	0.576876	0.376188	0.884628
GIF	0.011941	0.582207	0.381873	0.887637
VSTM2A	0.012051	0.578595	0.377458	0.886911
GLYATL2	0.013554	0.594046	0.392883	0.898208
HOXC13	0.016032	1.687068	1.102235	2.582206
ITLN1	0.017858	0.597059	0.389648	0.914874
GFY	0.017878	1.655044	1.09072	2.511341
CPN1	0.018671	0.605364	0.398446	0.919737
ITLN2	0.020521	0.611212	0.403	0.926995
GCG	0.021027	0.613834	0.40553	0.929133
DPCR1	0.021535	1.643644	1.075927	2.51092
GPR15	0.023106	0.609272	0.397329	0.934269
C19orf81	0.023211	1.619302	1.068034	2.455107
SLC38A8	0.025795	1.599945	1.058419	2.418535
REP15	0.029968	0.62727	0.411677	0.955768
ATOH1	0.0323	0.621861	0.402535	0.96069
HIST1H4F	0.033227	0.636932	0.420503	0.964756
HIST1H2BO	0.034221	1.577314	1.034477	2.405002
APOF	0.034271	0.639258	0.422431	0.967378
PAGE2	0.035262	1.561772	1.031263	2.365189
FTCD	0.037752	1.547991	1.025036	2.337749
UNC5D	0.040487	0.642471	0.420759	0.98101
PNLDC1	0.045544	1.534386	1.008522	2.334447
GPR83	0.049187	1.520442	1.001493	2.308298

Table S10. Gene signatures of nine immune cells list.

Immune cells	Gene list
Naïve T-Cell	CCR7,LEF1,TCF7,SELL
Effector T-Cell	CX3CR1,FGFBP2,FCGR3A
Effector memory T-Cell	PDCD1,DUSP4,GZMK,GZMA,IFNG
Central memory T-Cell	CCR7，SELL，IL7R
Resident memory T-Cell	CD69,ITGAE,CXCR6,MYADM
Exhausted T-Cell	HAVCR2,TIGIT,LAG3,PDCD1,CXCL13 LAYN
Resting Treg T-Cell	FOXP3,IL2RA
Effector Treg T-Cell	FOXP3,CTLA4,CCR8,TNFRSF9
Th1-like	CXCL13,HAVCR2,IFNG,CXCR3,BHLHE40,CD4
